# The Rotterdam Study: 2016 objectives and design update

**DOI:** 10.1007/s10654-015-0082-x

**Published:** 2015-09-19

**Authors:** Albert Hofman, Guy G. O. Brusselle, Sarwa Darwish Murad, Cornelia M. van Duijn, Oscar H. Franco, André Goedegebure, M. Arfan Ikram, Caroline C. W. Klaver, Tamar E. C. Nijsten, Robin P. Peeters, Bruno H. Ch. Stricker, Henning W. Tiemeier, André G. Uitterlinden, Meike W. Vernooij

**Affiliations:** Department of Epidemiology, Erasmus Medical Center, PO Box 2040, 3000 CA Rotterdam, The Netherlands; Department of Pulmonary Diseases, Erasmus Medical Center, Rotterdam, The Netherlands; Department of Internal Medicine, Erasmus Medical Center, Rotterdam, The Netherlands; Department of Gastro-Enterology, Erasmus Medical Center, Rotterdam, The Netherlands; Department of Cardiology, Erasmus Medical Center, Rotterdam, The Netherlands; Department of Otolaryngology, Erasmus Medical Center, Rotterdam, The Netherlands; Department of Neurology, Erasmus Medical Center, Rotterdam, The Netherlands; Department of Radiology, Erasmus Medical Center, Rotterdam, The Netherlands; Department of Ophthalmology, Erasmus Medical Center, Rotterdam, The Netherlands; Department of Dermatology, Erasmus Medical Center, Rotterdam, The Netherlands; Department of Psychiatry, Erasmus Medical Center, Rotterdam, The Netherlands

**Keywords:** Biomarkers, Cardiovascular diseases, Cohort study, Dermatological diseases, Endocrine diseases, Epidemiologic methods, Genetic epidemiology, Liver diseases, Neurological diseases, Oncology, Ophthalmic diseases, Otolaryngological diseases, Pharmacoepidemiology, Renal diseases, Psychiatric diseases, Respiratory diseases

## Abstract

The Rotterdam Study is a prospective cohort study ongoing since 1990 in the city of Rotterdam in The Netherlands. The study targets cardiovascular, endocrine, hepatic, neurological, ophthalmic, psychiatric, dermatological, otolaryngological, locomotor, and respiratory diseases. As of 2008, 14,926 subjects aged 45 years or over comprise the Rotterdam Study cohort. The findings of the Rotterdam Study have been presented in over 1200 research articles and reports (see www.erasmus-epidemiology.nl/rotterdamstudy). This article gives the rationale of the study and its design. It also presents a summary of the major findings and an update of the objectives and methods.

## Introduction

The Rotterdam Study was designed in the mid-1980s as a response to the demographic changes that were leading to an increase of the proportion of elderly people in most populations [1]. It was clear that this would produce a strong rise in elderly people living with diseases, as most diseases cluster at the end of life, and that to discover the causes of diseases in the elderly one would have to study risk factors of those diseases [2]. A major approach to finding causes is the prospective follow-up study, which has proven quite effective in finding causes of heart disease and cancer.

## The design of the Rotterdam Study

**Fig. 1:**
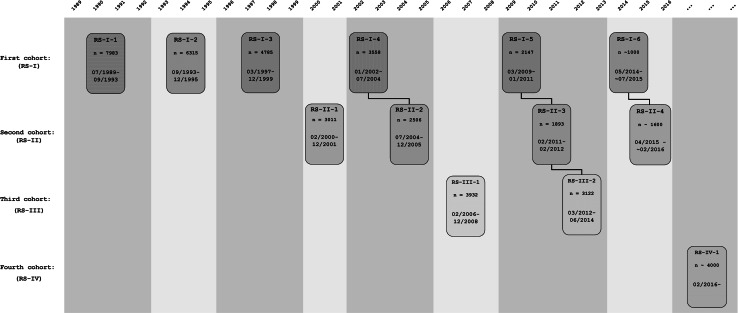
Diagram of examination cycles of the Rotterdam Study (RS). RS-I-1 refers to the baseline examination of the original cohort (pilot phase 07/1989–12/1989; cohort recruitment 01/1990–09/1993). RS-I-2, RS-I-3, RS-I-4, RS-I-5, and RS-I-6 refer to re-examinations of the original cohort members. RS-II-1 refers to the extension of the cohort with persons in the study district that became 55 years since the start of the study or those of 55 years or over that migrated into the study district. RS-II-2, RS-II-3, and RS-II-4 refer to re-examinations of the extension cohort. RS-III-1 refers to the baseline examination of all persons aged 45 years and over living in the study district that had not been examined already (i.e., mainly comprising those aged 45–60 years). RS-III-2 refers to the first re-examination of this third cohort. Examination RS-I-4 and RS-II-2 were conducted as one project and feature an identical research program. Similarly, examinations RS-I-5, RS-II-3, and RS-III-2 share the same program items. Also, examinations RS-I-6 and RS-II-4 are conducted as one project. RS-IV-1 refers to the baseline visit of a new cohort, to be established in February 2016

The design of the Rotterdam Study is that of a prospective cohort study among, initially, 7983 persons living in the well-defined Ommoord district in the city of Rotterdam in The Netherlands (78 % of 10,215 invitees). They were all 55 years of age or over and the oldest participant at the start was 106 years [3]. The study started with a pilot phase in the second half of 1989. From January 1990 onwards participants were recruited for the Rotterdam Study. Figure 1 gives a diagram of the various cycles in the study. In In 2000, 3011 participants (out of 4472 invitees) who had become 55 years of age or moved into the study district since the start of the study were added to the cohort. In 2006 a further extension of the cohort was initiated in which 3932 subjects were included, aged 45–54 years, out of 6057 invited, living in the Ommoord district. By the end of 2008, the Rotterdam Study therefore comprised 14,926 subjects aged 45 years or over [4, 5]. The overall response figure for all three cycles at baseline was 72.0 % (14,926 of 20,744).

The participants were all examined in some detail at baseline. They were interviewed at home (2 h) and then had an extensive set of examinations (a total of 5 h) in a specially built research facility in the centre of their district. These examinations focused on possible causes of invalidating diseases in the elderly in a clinically state-of-the-art manner, as far as the circumstances allowed. The emphasis was put on imaging (of heart, blood vessels, eyes, skeleton and later brain) and on collecting body fluids that enabled further in-depth molecular and genetic analyses. These examinations were repeated every 3–4 years in characteristics that could change over time. And so there were examination cycles from 1990 to 1993, from 1993 to 1995, from 1997 to 1999, from 2000 to 2001, from 2002 to 2004, from 2004 to 2005, from 2006 to 2008, from 2009 to 2011, from 2011 to 2012, from 2012 to 2014, from 2014 to 2015, and from 2015 onwards (Fig. 1). In January 2016 the fourth examination cycle for the second cohort (RS-II-4) will be finished. In February 2016 a fourth cohort will be established. The age range for this new cohort is predominantly 40–55 years, the anticipated number of participants is 4000.

The participants in the Rotterdam Study are followed for a variety of diseases that are frequent in the elderly: coronary heart disease, heart failure and stroke, Parkinson disease, Alzheimer disease and other dementias, depression and anxiety disorders, macular degeneration and glaucoma, respiratory diseases, liver diseases, diabetes mellitus, osteoporosis, dermatological diseases and cancer.

The Rotterdam Study has been approved by the institutional review board (Medical Ethics Committee) of the Erasmus Medical Center and by the review board of The Netherlands Ministry of Health, Welfare and Sports. The approval has been renewed every 5 years, as well as with the introduction of major new elements in the study (e.g., MRI investigations).

In the remainder of this article the objectives and major findings will be presented with an update of the research methods for cardiovascular diseases, dermatological diseases, endocrine diseases, liver diseases, neurological diseases, ophthalmic diseases, psychiatric diseases, respiratory diseases, as well as for genetic and biomarker studies and for pharmaco-epidemiologic studies. For relevant recent EJE references see [6–28].

## Cardiovascular diseases

### Objectives

Research on the epidemiology of cardiovascular disease focuses on the etiology, prognosis, and prediction of cardiovascular disorders (including coronary heart disease, stroke, heart failure) diabetes mellitus and metabolic syndrome. The main emphasis is on prevention and management of a first cardiovascular event but prevention of secondary events is also an area of interest. Putative risk factors include five groups: lifestyle factors, endocrine factors, factors involved in hemostasis, inflammation and endothelial function, metabolomic factors and genetic factors. We have five specific focused themes:

1. Lifestyle: focused on evaluating the role of lifestyle factors (including nutrition, physical activity, sleep and smoking) in maintaining cardiovascular health as well as the interactions that lifestyle factors might have on other factors (e.g. genes, epigenetic marks and medications).
2. Biomarkers and genes: aimed to identify relevant biomarkers for the identification of novel mechanisms of disease. These incorporate both molecular and genetic factors together with their potential interactions. Genomics, epigenetic marks and metabolomics play a key role.
3. Prediction and women’s cardiovascular health: aimed to improve the identification of individuals at increased risk of developing cardiovascular disease in order to point out windows of opportunities that could permit early preventive interventions and personalised care. A special focus is given to evaluating specific factors and formulating targeted strategies to prevent cardiovascular disease in women.
4. High risk: focused on predictors and prognosis of chronic cardiovascular conditions, like heart failure, pulmonary hypertension, and atrial fibrillation.
5. Imaging: this work theme aims to identify the contribution that new technologies can provide to the maximum benefit of early diagnosis and accurate prognosis. Major focus is on non-invasive assessment of atherosclerosis to improve the understanding of the atherosclerotic process and the prediction of cardiovascular disease, including measurement of coronary calcification with electron-beam and multi-detector CT (MDCT) and carotid plaque characterization by MRI.

### Major findings

#### Anthropometrics and cardiovascular disease

We evaluated different anthropometric measures, including body mass index, waist circumference, waist to height ratio, waist to hip ratio and a body shape index in association with all-cause, cardiovascular and cancer mortality. We have shown that among different anthropometric measures, body shape index was strongly associated with the risk of all-cause, cardiovascular and cancer mortality [29]. Within the European Network for Genetic and Genomic Epidemiology (ENGAGE) consortium, using a mendelian randomization approach, we found that adiposity, as indicated by body mass index, has a causal relationship with coronary heart disease, heart failure and for the first time, ischemic stroke [30]. Also, there were age- and sex-specific causal effects of adiposity on cardiovascular risk factors, including cholesterol, blood pressure, fasting levels of insulin and C-reactive protein [31].

#### Comparison of guidelines

The new American College of Cardiology/American Heart Association (ACC/AHA) guidelines introduced a new cardiovascular (CVD) prediction model and lowered the threshold for treatment with statins to a 7.5 % 10-year hard atherosclerotic cardiovascular disease (ASCVD) risk. Using 4854 asymptomatic participants from the population-based Rotterdam Study, we determined the implications of the new ACC/AHA guideline’s treatment threshold and risk prediction model and compared it with the Adult Treatment Panel III (ATP-III), and the European Society of Cardiology (ESC) guidelines. We showed that proportions of individuals eligible for treatment with statins differed substantially among the 3 guidelines [32]. The ACC/AHA guideline would recommend statins for nearly all men and two-thirds of women, proportions exceeding those with the ATP-III or ESC guidelines. All risk prediction models underlying the 3 guidelines provided poor calibration and moderate to good discrimination in our population. To facilitate better clinical decision making, improving risk predictions and setting appropriate population-wide thresholds are necessary.

#### Women’s health

Women experience multiple health issues throughout their life course differently from men. Therefore, attention to women’s health is important in all stages in life. To improve women’s quality of life and guarantee a long-lasting and active role for women in society, prevention of chronic diseases and disability is a key aspect. Our focus, therefore, in the women’s health group is on the major health issues for peri- and post-menopausal women, their risk factors, and prevention strategies [33].

As menopausal health is a crucial aspect in healthy and successful aging, we aimed to characterize a concept for healthy menopause. We conceptualized healthy menopause as a dynamic state, following the permanent loss of ovarian function, which is characterized by self-perceived satisfactory physical, psychological and social functioning, incorporating disease and disability, allowing the attainment of a woman’s desired ability to adapt and capacity to self-manage. Conceptualization of healthy menopause serves as a crucial step in improvement of health in menopausal women, allowing for adapting adequate preventive and treatment strategies [34].

Although cardiovascular disease (CVD) remains one of the leading causes of death and disability for both men and women, our research underscores considerable sex differences in the occurrence of the various manifestations of CVD. Using the long term follow-up from the prospective population based Rotterdam Study, we showed that despite similar lifetime risks of CVD at age 55 for men and women, considerable differences in the first manifestation exist. Men are more likely to develop coronary heart disease as a first event, while women are more likely to have cerebrovascular disease or heart failure as their first event, although these manifestations appear most often at older ages [35]. Since strategies for prevention of stroke and heart failure might differ from strategies for prevention of coronary heart disease, to devise a sex-tailored primary prevention program, knowledge about the first manifestation of diseases is important.

#### Heart failure and atrial fibrillation

The Rotterdam Study enabled accurate assessment of the incidence and lifetime risk of heart failure and atrial fibrillation in an elderly population [36–38]. It was shown that inflammation and resting heart rate is associated with risk of heart failure [39, 40]. In addition we identified several new risk factors of atrial fibrillation. We found that markers of generalized atherosclerosis in persons without a history of myocardial infarction or angina were associated with a higher risk of atrial fibrillation [41]. Furthermore, high-normal thyroid function [42] and higher levels of dehydroepiandrosterone sulfate, a precursor in the biosynthetic pathway of androgenic and estrogenic sex hormones were associated with incidence of atrial fibrillation [43]. In collaboration with several community-based prospective studies we were able to develop a prediction model for atrial fibrillation, only using variables that are routinely collected in primary care settings [44]. In a large collaborative study as part of the CHARGE consortium, we investigated the genetic variation responsible for 6 traits related to cardiac structure and function. We found two replicated loci for left ventricular dimension and 5 replicated loci for aortic root size [45]. Another topic of interest was the search for genetic determinants of several rhythm and conduction disturbances on the ECG, notably RR-interval, QRS duration, and QT(c)-interval, PR-interval, as well as atrial fibrillation and sudden cardiac death. For example, we identified several new loci for PR interval [46], heart rate [47], and atrial fibrillation [48, 49] in meta-analyses from the CHARGE consortium.

#### Cardiovascular risk factors and prediction

Endocrine, inflammatory and hemostatic factors and risk of coronary heart disease were addressed in several studies. Subclinical hypothyroidism was an independent risk factor of atherosclerosis and myocardial infarction in older women [50]. In a recent study, we compared the change in the accuracy of risk predictions when newer risk markers, representative of various pathophysiologic pathways, were added to the established clinical risk predictors. Among the biomarkers, improvements in coronary heart disease risk prediction were most significant with the addition of amino-terminal pro-B-type natriuretic peptide (NT-proBNP) [51, 52]. Furthermore, plasma C-Reactive protein (CRP) and lipoprotein-associated phospholipase A2 (Lp-PLA2) activity were independent predictors of coronary heart disease [53, 54]. Earlier findings included the association of tissue plasminogen activator (TPA) with incident coronary heart disease [55]. Using a comprehensive biomarker assay, we analysed multiple markers of inflammation among 800+ individuals with incident coronary heart disease [56]. We identified EN-RAGE as a novel biomarker for incidence of coronary heart disease, independent of established risk factors and inflammatory markers, such as C-reactive protein [56]. With respect to the prediction of coronary heart disease, EN-RAGE improved prediction significantly indicating that EN-RAGE might be useful in CHD prediction [56]. Recently, we developed and validated a coronary heart disease prediction model tailored for the aging population based on competing risk methodology [57]. Also, we have shown that the non-laboratory based model, based on body shape index, could predict risk of cardiovascular disease as accurately as one that relied on laboratory-based values among men [58].

#### Non-invasive measures of atherosclerosis

Multiple studies focused on the predictive value of non-invasive measures of atherosclerosis for risk of coronary heart disease. Strong associations with risk of coronary heart disease were found for carotid intima-media thickness [59], pulse wave velocity [60], and coronary calcification as assessed by electron-beam CT [61]. The relatively crude measures directly assessing plaques in the carotid artery and abdominal aorta predict coronary heart disease equally well as the more precisely measured carotid intima-media thickness [62]. In persons at intermediate risk of cardiovascular disease, coronary artery calcium provided the best increment in coronary heart disease risk prediction and stratification (to reclassify persons into more appropriate coronary risk categories) [51, 63, 64]. The burden of coronary calcification also provides incremental predictive information for heart failure, but nor for cerebrovascular disease [65, 66].

#### Genetic studies

Genetic studies included candidate gene studies [67] and more recently genome-wide association studies of clinical disease and risk factor phenotypes. So far we have contributed to more than 100 Genome-wide association (GWA) studies in the field of cardiovascular disease. These GWA studies are primarily conducted in the framework of the Cohorts for Heart and Aging Research in Genomic Epidemiology (CHARGE) Consortium [68, 69] however in many instances we include further studies. We identified 3 genetic loci associated with uric acid concentration and gout [70]. Three loss-of-function variants in HAL gene were found to associate with histidine levels [71] but not with coronary heart disease. We also identified a significant association between the UMOD gene which encodes Tamm-Horsfall protein and chronic kidney disease [72]. We found four genes for systolic blood pressure, six for diastolic blood pressure and one for hypertension [73–75]. We found multiple loci that influenced erythrocyte phenotypes in the CHARGE Consortium [76]. In a meta-analysis in more than 80,000 individuals from 25 studies, we identified 18 loci for CRP levels. The study highlighted immune response and metabolic regulatory pathways involved in the regulation of chronic inflammation [77]. Novel associations of 12 low-frequency exonic variants with plasma levels of factor VII, factor VIII, and von Willebrand factor were also detected [78, 79]). The association with these variants was independent of the previously identified common variants associated with these traits, and the effect sizes were larger. We performed the first GWA study of ADAMTS13 activity, identifying independent associations with three common variants at the ADAMTS13 locus, as well as one common variant at the SUPT3H locus [80]. Additionally, we used a genotyping array focused on rare exonic variants to identify three independent rare variants in the *ADAMTS13* gene associated with ADAMTS13 activity [80]. We have also identified genetic loci associated with the measures of subclinical atherosclerosis burden. Our genome-wide association studies on the 3 measures of subclinical atherosclerosis identified several new genetic loci [81–83]. We have contributed to GWA studies on coronary artery disease [84, 85]. Also, we found that 152 known coronary heart disease SNPs improved the prediction of prevalent but not incident coronary heart disease. This difference may be explained by biases related to the use of prevalent rather than incident coronary heart disease in genome-wide association studies [86]. In addition, by using genome-wide methylation data, we found an effect of tobacco smoking on DNA methylation of 12 coronary artery disease-related genes, and thus providing novel insights in the pathways that link tobacco smoking to risk of coronary artery disease [87].

Thus far, a large number of genetic variants have been identified by GWAS that contribute to the induction and development of cardio-metabolic diseases. Nevertheless, the vast majority of the identified variants map to the non-coding regions of genome that their biological relevant to the disease remain unclear. Non-coding RNAs play regulatory roles in various biological processes and cellular contexts. We identified a number functional variants in microRNA-genes and microRNA binding sites on the 3ÚTR of coding genes that affect miRNA gene regulation and explain some of the observed associations from GWAS of cardio-metabolic phenotypes [88–90].

#### Nutrition and lifestyle

We found that subjects who had fish intake of more than 19 g/day had a significantly lower prevalence of mild/moderate coronary calcification [91]. Also, we found that an increase of 50 g in processed meat was associated with increased CRP levels [92]. In addition to this, the intake of processed meat was associated with a higher risk of type 2 diabetes which was independent of CRP levels [92]. The dietary intake of fatty acids and its relation with CRP levels was studied as well. We found that higher intake of total PUFAs was associated with lower CRP levels and the intake of n-6 PUFAs were inversely associated with CRP [93]. We did not observe an association between n-3 PUFAs or n-3:n-6 PUFA ratio and CRP. Likewise, we studied whether dietary proteins, amino acids and acid load were associated with the risk of hypertension. It appeared that these factors were not a major determinant of the risk of hypertension in the Rotterdam Study [94–96]. On the other hand, we found that higher vitamin D concentrations were associated with lower prevalence of metabolic syndrome. In particular, adequate levels of vitamin D were associated with more beneficial HDL, triglycerides, waist circumference and serum glucose levels [97]. We did not observe an association between vitamin D and atrial fibrillation [98]. Also, we found no association between coffee consumption and incident dementia [99]. High-fat dairy was inversely related to fatal stroke, but not to incident stroke. Total dairy consumption or dairy subgroups (e.g. milk, low-fat dairy) were not associated with the occurrence of CVD events [100]. We investigated relationship of epicardial fat volume with atherosclerotic calcification volume and found that larger epicardial fat volume was related to coronary and extracranial carotid artery calcification volume in males only [101]. As part of the CHANCES consortium, we found that adherence to a healthy diet according to the WHO was associated with greater longevity [102].

Besides main effects of nutrition, we studied gene-nutrient interactions. We found that dietary vitamin E intake may modulate the relation of SIRT1 genetic variants with body mass index [103]. Also, we studied the modification of magnesium, whole grain and a healthy diet score on fasting glucose and insulin by SNPs related to fasting glucose and insulin as part of the CHARGE consortium [104–106]. Furthermore, low dietary carbohydrate intake and normal sleep duration were found to ameliorate cardiometabolic abnormalities conferred by common circadian-related genetic variants [107].In addition to nutrition, we also investigated lifestyle factors and found that there is no association between positive psychological well-being and incidence of cardiovascular disease [108]. We did find an association between clinical heart failure and poor sleep quality over time, but did not find an association between cardiac dysfunction (measured by echocardiography) and sleep quality [109].

### Methods update

#### Clinical follow-up

Information on clinical cardiovascular outcomes is collected through an automated follow-up system. The follow-up system involves linkage of the study base to digital medical records from general practitioners in the study area and subsequent collection of letters of medical specialists and discharge reports in case of hospitalisation. With respect to the vital status of participants, information is also obtained regularly from the municipal health authorities in Rotterdam. After notification, cause and circumstances of death are established by questionnaire from the treating physicians. Clinical cardiovascular outcomes are adjudicated according to established definitions based on international guidelines by study physicians and medical specialists in the field affiliated with the Rotterdam Study. Methods of follow-up data collection, adjudication of events, and definitions of cardiovascular end points have been described in detail previously in this journal [110]. Systematic follow-up data collection is done for the occurrence of cardiovascular mortality, coronary heart disease (including coronary death, myocardial infarction, and coronary revascularization procedures), heart failure, atrial fibrillation, and sudden cardiac death [110]. Diabetes mellitus is defined based on guidelines of the American Diabetes Association and the World Health Organization. We defined incident diabetes as fasting plasma glucose level ≥7.0 mmol/L, or the use of oral antidiabetic medication or insulin, or treatment by diet and registered by a general practitioner as having diabetes.

#### Cardiovascular risk factors

Besides traditional cardiovascular risk factors, five major groups of putative risk factors for cardiovascular conditions are examined. The first group are lifestyle factors, including dietary factors, physical activity, smoking, sleep and vitamin D (as described above). The second are endocrine factors, including diabetes, sex hormones, thyroid gland and adrenal gland hormones and natriuretic peptides (e.g. [42, 43, 50–52]). The third group comprises factors involved in hemostasis, inflammation and endothelial function (e.g. [53, 111, 112]). The fourth group covers genetic factors. In addition to the candidate gene approach, studies are more recently conducted through the genome-wide association approach (e.g. [45–49, 70, 72–77, 81–85, 104–106, 111]). In genome-wide association studies, data from the Rotterdam Study are often combined with those from other studies in the context of the large collaborative CHARGE consortium [68, 69]. Within the fifth group we are applying both proton nuclear magnetic resonance (^1^H NMR) and mass spectrometry (MS) for metabolic profiling in 2000 participants of the Rotterdam Study including nearly 200 incident cases of coronary heart disease. Furthermore, in this context, special attention has been given to the contribution of different risk factors in relation to cardiovascular disease in women. Data has been collected to evaluate the impact of specific periods of potential vulnerability across a woman’s lifespan; menarche, pregnancy, and menopause. Also, DNA methylation can regulate gene expression without altering the underlying DNA sequence and is now emerging as a promising molecular strategy for risk stratification for complex disease, including cardiovascular disease. Using the Illumina Infinium HumanMethylation450 array, we have generated DNA methylation profiles of ~480,000 CpG sites in In ~1000 samples of the RS-III.

#### Non-invasive measures of atherosclerosis

At baseline and follow-up examinations, ultrasonographic assessments of carotid intima-media thickness and carotid plaques were conducted in all participants [59]. At these examinations, also measurements of the ankle-brachial index and aortic calcification (on X-rays of the lumbar spine) were obtained [62]. Carotid–femoral pulse wave velocity, a measure of aortic stiffness, was measured in all participants of RS-I-3, RS-II-1, and RS-III-1 with an automatic device [60]. Measurements of coronary calcification by electron-beam CT and more recently by MDCT were conducted from 1997 onwards in RS-I and RS-II [61], [63]. From 2003 to 2006, MDCT was used to also quantify calcification in the aortic arch and carotid arteries in RS-I and RS-II. Measurement of carotid plaque components using MRI was done from 2007 to 2012 in all participants from RS-I, RS-II and RS-III with carotid wall thickening on conventional carotid ultrasound. Repeated MRI measures over time were obtained in RS-I and RS-II.

#### Electrocardiographic, echocardiographic and other ultrasound measurements

At every exam, a 12-lead 10-s resting ECG is made and processed by the Modular ECG Analysis System (MEANS) to obtain a series of ECG measurements [113]. Abdominal aortic diameters were measured by ultrasound at RS-I-1, and from 2002 (RS-I-4) onwards in all three Rotterdam Study cohorts. Also from 2002 onwards (RS-I-4), repeated echocardiographic measurements are conducted of structural and functional left heart parameters [114]. From 2009 (RS-I-5) onwards, measurements of structure and function of the right heart are also collected, including estimates of pulmonary artery pressure. In the same round a 3-min resting ECG was measured in all participants.

#### Nutrition and lifestyle

Nutritional data has been collected in RS-I-1, RS-I-5, RS-II-1, RS-II-3 and RS-III-1 by using semi quantitative food frequency questionnaires (FFQ). In RS-I-1 and RS- II-1, participants completed a checklist about foods and drinks they had consumed at least twice a month during the preceding year and a standardized interview using a validated 170-item semi-quantitative FFQ [115]. In RS-I-5, RS-II-3 and RS-III-I, a more comprehensive FFQ was used during the visits as described in detail previously [116–119]. Development and processing of nutrition data is being performed in close collaboration with the Department of Human Nutrition, Wageningen University, the Netherlands. In RS-I-III, RS-I-5, RS-II-I-3 and RS-III-I, physical activity data was assessed by means of an adapted version of the Zutphen Physical Activity Questionnaire and the LASA Physical Activity Questionnaire [120–122]. The questionnaire contained questions on walking, cycling, gardening, diverse sports, hobbies and on housekeeping. According to time spent in light, moderate and vigorous activity, metabolic equivalents of task were calculated. Furthermore, we are implementing objective measurement of physical activity with triaxial accelerometers in all participants.

For additional EJE references concerning cardiovascular disease see [123–182].

## Dermatological diseases

### Objectives

Dermatoepidemiologic research in the Rotterdam Study focuses on the frequency of the most common skin conditions as well as on genetic and environmental factors associated with these skin diseases. The emphasis is on cutaneous malignancies such as basal and squamous cell carcinomas (BCC and SCC, respectively) and their precursor lesions (actinic keratosis), inflammatory dermatoses such as eczema and psoriasis, and varicose veins. Also, we examine the frequency and determinants including genetics and environmental exposures of skin aging (pigmentation, wrinkling and photodamage) and other visible traits in collaboration with the department of Genetic Identification. Recently, we have introduced optic measures of UV exposed and non-exposed to assess whether they can function as biomarkers of skin and internal diseases.

### Methods

In 2010, dermatology studies were introduced in the Rotterdam Study. To the home interview several items have been added questioning ultraviolet light exposure, history of (personal and familial) psoriasis, history of skin cancer, the diagnostic criteria of British Association of Dermatology for atopic eczema, adjusted diagnostic criteria for psoriatic arthritis. More recently, items on skin care and seborrheic dermatitis/dandruff were added.

A full body skin examination by physicians trained in dermatology with a focus on the most common skin diseases is the core contribution of dermatology. The clinical presence and extent of specific skin diseases (i.e., actinic keratosis, malignancies, psoriasis, seborrheic dermatitis, xerosis, hand and flexural eczema, alopecia, and signs of chronic venous insufficiency based on the ‘C’ of the CEAP classification) at time of examination is assessed in a standardized fashion. Other dermatological diseases will just be noted.

The extent of skin aging as a global score and broken down in different aspects such as wrinkling, pigmentary spots, and teleangiecatsia are scored using a validated photonumeric scales and computer algorithms. The Norwood–Hamilton classification and the Ludwig classification is used for male and female pattern hair loss, respectively. Fully standardized 3-dimensional photographs (Premier 3dMDface3-plus UHD, Atlanta, USA) of the face are taken to further assess skin characteristics including sagging, wrinkling at different sites, teleangiectasia and pigmented spots. The colour of the facial skin and at the inner side of the upper arm are measured using a spectrophotometer (Konica Minolta Sensing, spectrophotometer CM-700d, Singapore). Recently, we have introduced optic measures of UV exposed and non-exposed skin such as relectance and fluorescence spectroscopy including the AGE reader.

As for other cancers, pathology data of the cutaneous malignancies is obtained from linkage to the national cancer registry and the Dutch pathology database (PALGA). In a further attempt to identify cohort members with psoriasis, medical files and dispenses at pharmacies have been investigated resulting in over 350 psoriasis cases.

In 2016, we aim to start collecting skin microbiome in a randomized manner among all participants. For selected skin diseases such as eczema, rosacea, seborrheic dermatosis and psoriasis the affected skin will be sampled as well.

### Major findings

In the first follow-up study including the skin examinations of more than 2000 cohort members, showed that actinic keratosis is very common in this elderly population (AK prevalence was 49 % for men and 28 % for women) [183]. After adjusting for other factors, baldness in men was associated with a strongly increased risk of actinic keratosis.

A recent update yielded more than 1500 participants with a history of BCC, 450 with a SCC and 150 with a melanoma. We have demonstrated that approximately 30 % of people with a BCC develop multiple tumors with 5 years and have developed a prediction model to identify these high risk patients. A first genetic analysis could not confirm any of the existing BCC polymorphisms to be associated with the development of multiple BCC [184]. In collaboration with researchers from the Nurses Health Study, Framingham Heart Study and deCODE, the association between common genetic variants and these skin cancers is currently being examined.

Recently, we have presented the first GWAS on actinic keratosis [185]. Several skin color genes such as IRF4, MC1R, ASIP and BCN2 were significantly associated with these premalignant skin lesions independently from skin color.

In a candidate gene study in almost 6000 people, we confirmed known and identified new variants associated with digital skin colour extraction. Of the two new skin color genes, the genetic variants in UGT1A were significantly associated with hue and variants in BNC2 were significantly associated with saturation [186]. In the International Visible Trait Genetics Consortium, we identified novel pigmentation genes confirmed by functional follow up [187]. Several pigmentation genes were also significantly associated with the presence of pigmented facial spots in a GWAS [185].

The psoriasis patients within the Rotterdam Study have predominantly mild disease. The distribution of subclinical artherosclerosis measures as well as the cardiovascular events were comparable between the 262 psoriasis patients and the reference population [188]. However, psoriasis patients were significantly more likely to have signs of nonalcoholic fatty liver disease based on ultrasonography than their controls after adjusting for potential confounders [189]. Moreover, psoriasis patients were more likely to have liver fibrosis than controls comparing Fibroscan data [190].

## Endocrine and locomotor diseases

### Objectives

The main objective of the programme of endocrine and locomotor epidemiology research is to study frequency and etiology of major disorders of the endocrine glands (pituitary, reproductive, thyroid, parathyroid, adrenal, and neuro-endocrine pancreas) and the musculoskeletal system and their risk factors. These include diabetes mellitus, osteoporosis, osteoarthritis, reproductive traits (fertility, age-at-menopause), and hypo- and hyper-thyroidism. The evaluation of risk factors for the above mentioned conditions includes serum measurements (such as classical hormones and other endocrine molecules), imaging of bones and joints by DXA, X-ray and pQCT, measuring muscle strength, and genetic determinants of endocrine diseases and traits. In addition, consequences of these endocrine disorders are studied in relation to aging related diseases, including cardiovascular disease, diabetes, eye diseases, skin diseases, and neurocognitive decline.

### Major findings

In the process of obtaining digitized X-rays for all participants at all time-points of follow-up, we have evaluated the 3 major methods to score vertebral fractures: quantitative morphometry, semi-quantitative morphometry, and the qualitative ABQ method [191]. Prevalence of vertebral fractures differed substantially by the different methods, so standardization is crucial for patient care and for large scale epidemiological studies. Using the digitized X-ray scores, we have for the first time determined population-based prevalence of Scheuermann’s disease in the Dutch population to be 4 % [192].

In the relationship of type 2 diabetes with bone health we observed that diabetic subjects with inadequately controlled glucose control had 1–5 % increased bone mineral density (BMD) and ~50 % increased fracture risk, compared to diabetics with adequately controlled glucose and to non-diabetics [193].

By studying bone health across different types of hip osteoarthritis (OA), we observed that subjects with atrophic OA (i.e., with joint space narrowing but without osteophytes) have ~50 % increased fracture risk as compared to controls without OA [194]. In addition, we found that hip geometry measures had modest ability to predict hip OA, in addition to clinical risk factors [195].

Over the last years, we have scored X-ray all radiographs of knee, hip and hand of RS I, II and III for osteoarthritic features including up to 20 years of follow-up radiographs. In addition, we have (bilateral) knee MRI images available for a subset (±1000) individuals of RS III, including a longitudinal follow-up MRI after 6 years. In addition, pain sensitivity measurements have been performed including a quantitative assessment of heat sensitivity on the arm using a standardized device (TSA-II neurosensory analyzer, Medoc), and indications of (wide-spread) pain in any part of the body using a manikin. This deep musculoskeletal phenotyping makes the Rotterdam Study a unique resource to study determinants of osteoarthritis (OA) and chronic pain and is the largest such dataset in the world. We have developed and validated (internally and externally) a prognostic model for knee osteoarthritis using clinical, genetic and biochemical risk factors [196]. We demonstrated that CTXII is the most informative biomarker for prediction of OA, while two other well-known biomarkers (COMP and C2M) are only consistently associated with prevalence of OA [197]. This indicates that COMP and C2M are merely descriptive of current OA-activity, while CTX2 has additive value for prediction of the course of disease.

We have identified associations between the presence of OA and CVD, i.e., measures of atherosclerosis [198]. However, in a longitudinal design, we observed that participants with radiographic OA were not at increased risk of CVD. Our results suggest that the close relation between disability and osteoarthritis may explain previous reports suggesting a relationship between OA and CVD [199].

Discordance between having pain and radiologic OA is a well-established fact. We have identified several factors associated to pain, which were not related to structural damage of the joints [200]. We demonstrated that type 3 finger length pattern, an indicator of prenatal androgen exposure, was associated with having symptomatic knee OA, and chronic pain [201].

We demonstrated that high-normal thyroid function is associated with an increased risk of atrial fibrillation [42]. In addition, in the first study to investigate the association between thyroid function and age-related macular degeneration (AMD), we observed an increased risk of AMD with higher values of thyroid hormone, even in the normal range of thyroid function [202]. We also for the first time identified lower TSH levels within the normal range as a risk factor for developing depression in the elderly [203].

The research line endocrine diseases is also actively involved in collaborating with economist to study the biology of economic behaviour, in particular entrepreneurial behaviour (and related traits such as educational attainment). In one of the first collaborative analyses we found no evidence for a relationship of testosteron levels with entrepreneurial behaviour [204].

Much of the work of this research is made possible by large-scale collaboration in consortia, some of which focus on one particular disease or trait (e.g., GEFOS), while others are more broad spectrum strategic collaborations (e.g., CHARGE, ENGAGE). We are part of several such large consortia studying genetic and epidemiological risk factors for osteoporosis (GEFOS, GENOMOS, CHANCES, CHARGE), osteoarthritis (TREAT-OA, ArcoGen), diabetes (MAGIC), thyroid disease (CHARGE and Thyroid Studies Collaboration (TSC)), anthropometric (GIANT) and reproductive traits (CHARGE, REPROGEN, PCOSGEN).

As part of the TSC, we recently published three individual-participant data analyses. By analyzing individual participant data from 13 prospective cohorts (70,298 participants) we demonstrated that subclinical hyperthyroidism is associated with an increased risk of hip and other fractures, particularly among those with TSH levels of less than 0.10 mIU/L and those with endogenous subclinical hyperthyroidism [205]. An analysis combining data from 17 cohorts and lead by the Rotterdam Study did not show a higher risk of stroke with subclinical hypothyroidism except in participants younger than 50 years of age [206]. A combined analysis in 14 cohorts focusing on risk of coronary heart disease showed no relationship of TSH levels within the reference range and risk of CHD events or CHD mortality [207].

### Major GWAS findings

With >82,000 samples collected within the GEFOS consortium a landmark publication was achieved with the identification of 56 loci influencing bone mineral density in total explaining ~5 % of genetic variation in BMD, and of which 14 loci also were associated with fracture risk [208]. A large GEFOS meta-analysis is currently underway to identify risk factors for fracture per se, including all types of fracture, hip fracture and vertebral fracture.

In a meta-analysis of 15,000 hip OA cases and 54,000 controls assembled in the arcOGen consortium, 5 novel loci influencing risk of hip OA were identified [209]. In an analysis of a so-called endo-phenotype of OA, i.e., joint space width narrowing (JSN) a GWAS among 10,000 subjects we identified a novel locus, called DOT1L, to influence cartilage thickness, as well as the risk for hip OA [210]. Interestingly, this gene product is a known drug target for treating leukemia with several drugs under development. Our group led a large scale meta-analysis to chronic wide spread pain in 2700 cases and 14,000 controls and identified for the first time a genetic locus involved in pain, i.e., CCT5 [211]. Within the TREAT-OA consortium, we assessed the effect of genetic variants of 199 OA candidate genes and identified a genetic variant in COL11A1 to be associated with hip OA [212]. In addition, we identified genetic loci affecting either uCTX-II or sCOMP levels, two biochemical markers for OA [213] and a novel locus for hand OA [214].

In the largest meta-analysis of GWAS of age-at-menopause among 62,000 women and which our group led, we identified 13 loci associated with differences in age-at-menopause and explaining ~5 % of the genetic variation [215]. Interestingly, the majority of the loci most likely involve genes involved in the DNA repair pathway which points to the importance of this system in maintaining an error-free stemcell lineage to which the oocyte belongs. As such the phenotype of age-at-menopause, represents an interesting model for age-related changes in cell function maintenance and functions as a model to identify molecular mechanisms for damage accumulation and repair during ageing.

In a meta-analysis of GWAS data on TSH levels and free T4 levels derived from up to 26,000 subjects, 26 loci were identified explaining 2–5 % of the genetic variation of TSH and fT4 respectively [216]. There was only limited overlap between the loci for TSH and fT4, and evidence was obtained for 5 loci to have sex-specific effects. A GWAS meta-analysis focusing on TPO autoantibodies (an important clinical marker for the detection of early AITD) in 16 cohorts identified five newly associated loci, three of which were also associated with clinical thyroid disease. With these markers we identified a large subgroup in the general population with a substantially increased risk of TPOAbs [217]. A follow-up study identifying 4 additional loci associated provided further insight into the genetic underpinnings of hypothyroidism. A Genetic Risk Score showed strong and graded associations with markers of thyroid function and disease in independent population-based studies [218].

An interesting GWAS involved the analysis of Helicobacter pylori serologic status among members of the Rotterdam Study and the SHIP cohort [219]. Two novel loci were identified, TLR1 and FCGR2A, which can help explain inter-individual differences in risk for H pylori infection. A larger meta-analysis including several CHARGE cohorts is currently underway.

For many endocrine biomarkers GWAS have been performed to identify the genetic loci influencing their serum levels (e.g., homocysteine, testosteron, SHBG, thyroid hormone levels, etc.) and these are also involved in several mendelian randomization analyses in relation to major disease endpoints for which these biomarkers have been suggested to be predictive.

### Methods update

For all participants DXA-based bone mineral density (BMD) measurements of the lumbar spine, dual hip and total body BMD, as well as determination of body composition parameters are assessed with a ProdigyTM total body fan-beam densitometer (GE Lunar Corp, Madison, WI, USA). Hip structural analysis (HSA) of DXA scans including hip strength indexes (using software by GE Lunar) is determined for all scans. All lumbar spine measurements have been scored for the trabecular bone score (TBS), Since 2009 we perform iDXA measurements (GE Lunar) which measures L1–L4 BMD, bilateral total hip and femoral neck BMD and total body BMD. From the total body scan, we measure lean mass and fat mass body composition, including total body, trunk, arm, legs, and android and gynoid regions of interest. X-ray examinations of vertebral bodies, hips, knees and hand/wrist are since 2009 obtained by a digitalized Fuji FCR system (FUJIFILM Medical Systems) for all participants. Analogue X-ray photographs from previous time—points at all follow up measurements (~75,000 X-rays) have now all been digitized. All the X-rays have now been completely assessed for the presence of vertebral fractures and/or degenerative changes of the different joints (e.g., osteoarthritis, Scheuermann’s Disease). Vertebral fractures and deformities are assessed using the classical quantitative McCloskey–Kanis method, the semi-quantitative Genant method, and a qualitative algorithm-based technique termed the ABQ method. Incident clinical fractures (of all bone sites) are obtained from computerized records of the general practitioners and hospital registries which are regularly checked by research physicians who review and code the fracture information. Peripheral Quantitative Computarized Tomography (pQCT—Stratec XT2000) is a low-radiation technology (low radiation dose <1 uSV), which allows measuring structural properties of the bone and muscle cross section is now applied at the distal and proximal tibia (weight bearing). Measurements include geometrical parameters of volume, density of cortical and trabecular compartments and dimensions of bone and muscle. The test is done at the proximal tibia.

Muscle strength is assessed in all participants with a hand grip dynamometer. In addition jumping mechanography (Leonardo Novotec medical) has been implemented for all participants. This platform permits the dynamic examination of muscle contraction and power. In young individuals the test is performed asking participants to execute 2–3 counter movement two-footed stand (jumps) until both legs are straight and arms moving freely after receiving the instruction. The test is implemented as the “stand from a chair test” in subjects older than 85 years or with physical limitations. Muscle mass estimates are derived from whole body iDXA measurements.

The incidence and progression of OA is assessed using Kellgren scores obtained from X-rays of hips, knees, hands, and spine. The complete set of X-rays (all participants, all follow-up time points) has now been evaluated for the Kellgren score at these 4 joints. Novel diagnostic assessments for OA are available using Magnetic Resonance Imaging (MRI) of the knees in a large subset of the population (n = ~1000 RS-III). In addition, pain measurements were added in 2011 in this research line including a quantitative assessment of heat sensitivity on the arm using a standardized device, and indications of (wide-spread) pain in any part of the body using a pain puppet.

Advanced Glycation End products (AGEs) are glyco-oxidation products that accumulate in the body over a lifetime as part of normal ageing but formation is increased under hyperglycemic and oxidative circumstances. AGE accumulation in long-lived tissues may be associated with ageing of multiple organs and with risk to develop multiple chronic diseases Advanced glycation end-products in the skin are measured in RS-III-2, RS-I-5 and RS-II-3 by skin autofluorescence using an AGE-Reader™ (DiagnOptics B.V., Groningen, The Netherlands). These data are currently analyzed in relation to several diseases.

Several specific biomarker assessments in blood/serum/plasma and urine are done for the diagnosis and evaluation of risk factors of endocrine and metabolic diseases (e.g., glucose, TSH, freeT4, steroid hormones, Vitamin D, calcium, phosphate, CTXII, etc.). Fasting blood samples are collected along with challenged samples as part of a glucose tolerance test. Saliva is collected before and after a dexamethasone-suppression test. Finally, validated questionnaires evaluating nutrient intake (e.g., calcium and vitamins) and activities of daily living, allow to evaluate the role of environmental factors in endocrine conditions and locomotor diseases of the elderly.

For additional EJE references concerning endocrine and locomotor diseases see [220–242].

## Liver diseases

### Objectives

Fibrogenesis of the liver is most probably not only the result of well known liver diseases, such as viral hepatitis, alcoholic liver disease or non-alcoholic fatty liver disease (NAFLD), but rather a complex interaction between a genetic predisposition and these liver disorders. Liver research in the Rotterdam Study will concern the association between these known causes of liver disease and the occurrence, magnitude, and progression of fibrosis in combination with genetic and environmental factors. Additional research focus is on NAFLD. NAFLD is considered the hepatic manifestation of the metabolic syndrome and has become the most common chronic liver disease in Western countries in parallel with epidemics of obesity and type II diabetes mellitus. We aim to study the occurrence and risk factors of NAFLD in a general population and generate insight into the association with cardiovascular morbidity and mortality.

### Methods

#### Abdominal ultrasound

From February 2009 onwards (cohorts RS-I-5, RS-II-3 and RS-III-2) onwards, trained technicians perform abdominal ultrasonography in Rotterdam Study participants. Liver, biliary tract, gall bladder, spleen, pancreas, and kidneys in combination with doppler examination of hepatic veins, hepatic artery and portal vein will be evaluated. All images are stored digitally and will be reevaluated by an ultrasound trained physician.

#### Assessment of steatosis

The diagnosis and grading of liver steatosis is based on ultrasonographic liver brightness, hepatorenal echo contrast, deep attenuation and vessel blurring [243].

Non-alcoholic fatty liver disease is diagnosed by presence of steatosis on ultrasound and exclusion of excessive alcohol consumption, presence of viral hepatitis, use of known fatty liver-inducing pharmacological agents, recent bariatric surgery and a history of inflammatory bowel disease.

#### Assessment of fibrosis

Ultrasonographic evaluation of the liver parenchyma and liver surface is performed in order to assess severe fibrosis and/or cirrhosis. Additionally, sonographic signs of portal hypertension will be studied (splenomegaly, venous collaterals, portal vein diameter and flow, hepatic venous flow, and the presence of ascites).

To assess and quantify the grade of fibrosis trained technicians perform transient elastography in all participants by the Fibroscan. This test measures non-invasively and quantitatively the liver stiffness using a probe which includes an ultrasonic transducer transmitting a vibration wave through the liver. The velocity of the ultrasonic wave correlates directly with tissue stiffness [244, 245].

### Determinants of interest

The association between factors known to influence liver function and the occurrence of steatosis and fibrosis are being studied. Additionally the association of these conditions with age, gender, nutritional intake, concurrent alcohol intake, risk factors for viral hepatitis, BMI, waist-to-hip ratio, serum glucose, insulin, and diabetes mellitus, serum cholesterol and triglycerides are investigated. All clinical information is obtained by interview (updated with liver specific questions) and clinical examination. More recent efforts are focused on identifying common genetic variants associated with liver steatosis and/or fibrosis.

### Main findings

We found a high prevalence of NAFLD of 35.1 % within the Rotterdam Study population [246]. Main risk factors for NAFLD were found to be age, decreased physical activity lever, smoking, increased waist circumference, glucose intolerance, hypertension, and hyperlipidemia. Inversely, the risk of NAFLD seems to decrease after statin therapy [247]. Furthermore, using our ultrasound data as reference, we examined the performance of the well-known fatty liver disease index (FLI, based on waist circumference, BMI, triglyceride and gamma-glutamyltransferase (GGT) levels) in the Rotterdam Study population, and found that the FLI is a highly valid tool to predict NAFLD [248]. In another study, we found that all serum liver enzymes are related to all-cause mortality, as well as specifically cardiovascular (GGT) and cancer-related (alkaline phosphatase and aspartate aminotransferase) mortality [249]. More recently, we have examined the role of genetic factors in the multifactorial etiology of liver fibrosis, and found for example that the single nucleotide polymorphism (SNP) of the interferon gamma receptor 2, a pro-inflammatory gene known to be associated with progression to liver fibrosis in chronic hepatitis C patients, also was related to liver stiffness in the Rotterdam Study participants [250]. More studies are currently underway to look at other known and unknown genetic factors.

## Neurological diseases

### Objectives

Neuroepidemiologic research in the Rotterdam Study focuses on the frequency, etiology and early recognition of the most frequent neurologic diseases in the elderly. We study neurodegenerative diseases (dementia, including Alzheimer disease and Parkinson disease), cerebrovascular disease (both ischemic stroke and intracerebral hemorrhage), migraine and polyneuropathy. In all of these disorders clinical symptoms typically become manifest late in the disease course, the occurrence of clinical disease does not reflect the underlying spectrum of disease-related pathology, and most of the clinical syndromes are etiologically heterogeneous. Therefore, an additional research focus is on the causes and consequences of pre-symptomatic brain pathology that can be assessed with non-invasive modalities, which include MR-imaging, cognitive testing, gait assessment, and electromyography (EMG).

### Major findings

Neurodegenerative and cerebrovascular diseases are highly frequent in the elderly. The prevalence increases from age 55 to 65 years to age 90 years and above from less than 1 % to over 40 % for dementia [251], from less than 0.5 % to more than 4 % for Parkinson disease [252], and from approximately 1 % to nearly 10 % for stroke. The incidence figures follow this pattern of a strong increase with age over the entire age range, with the age-specific incidence of dementia being identical for men and women at least until the age of 85 [253] but with men having a higher age-specific incidence of both stroke and Parkinson disease than women throughout the age range [254, 255]. However, more recent numbers from 2000 onwards suggest that the relative incidence of dementia and stroke may be lower than in the 1990s [256–258]. Still, in absolute numbers these disease will dramatically increase in prevalence over the coming decades.

Vascular pathology and vascular risk factors are associated with worse cognitive performance [259], which also translates in people with vascular pathology or risk factors for vascular disease having an increased risk of dementia, including Alzheimer disease [260, 261]. Moreover, several life style factors are associated with the risk of dementia and Alzheimer disease [262, 263], suggesting that onset of dementia may at least partly be delayed or prevented. However, many of these lifestyle factors have only a short-term effect, suggesting reverse causality to some extent [264]. Commonly used drugs may also have a role in development of dementia [265]. Similar risk factor profiles also underlie cognitive decline prior to the clinical diagnosis of dementia [266, 267]. We recently investigated the total contribution that known modifiable risk factors make to total dementia burden and compared it across decades. We found that around 30 % of all dementia could be prevented by effectively treating known risk factors and that this percentage had remained stable across the last two decades.

The classical risk factors for stroke also associate with the risk of stroke in the Rotterdam Study [268–271]. We have also recently shown emerging risk factors to associate with stroke [206]. We have also developed prediction rules for ischemic and hemorrhagic stroke separately [272]. Important to note is that a large amount of stroke goes clinically undetected [273]. Nearly 20 % of elderly people have at least one silent brain infarct, and thereby a nearly fourfold increased risk of clinical stroke, a more than doubled risk of dementia including Alzheimer disease, and an increased risk of depression [274].

More recent work in the Rotterdam Study has been focusing on showing direct links between clinical cerebrovascular disease and dementia. In this regard, we showed subclinical heart disease to increase the risk of both stroke and dementia [275], intracranial carotid calcification to relate to both diseases [276], and subjective memory complaints to indicate a strongly increased risk of stroke [277]. With the advent of genome-wide association studies, the Rotterdam Study has contributed to large-scale collaborations and contributed to the identification of novel genes underlying the risk of Alzheimer disease and stroke [278–282].

We have also shown that although currently known genetic variants for dementia associate with cognition in non-demented elderly, these do not improve prediction [283].

Neuroimaging reveals that brain pathology is widespread [284] and can go clinically undetected for a long time. In addition to the silent infarcts, many apparently healthy elderly have ischemic changes in their cerebral white matter, i.e. white matter lesions, that are associated with an increased risk of dementia, stroke and depression. Also brain atrophy, especially of the hippocampus, is already present years before onset of even the earliest sign of cognitive impairment or subjective complaints. This emphasizes the need to shift the attention in etiologic research of neurodegenerative and cerebrovascular disease to the causes of pre-symptomatic and underlying brain changes. Technological advances in image acquisition, optimized imaging sequences and automated post-processing of multispectral MR data are major drivers of the rapid developments in this field. With our current imaging protocol we can now not only investigate established markers of brain pathology, such as infarcts, white matter lesions, and atrophy, but also extend towards novel markers, such as cerebral microbleeds and diffusion tensor imaging [285]. We have shown that risk factors profiles for subclinical disease overlap with those for clinical disease [280, 286–290]. Also, subclinical white matter lesions and silent infarcts improve the clinical prediction of incident stroke [291]. The clinical relevance of subclinical pathology is demonstrated by strong associations of these markers with morbidity and mortality [283, 292–295]. We have also extensively studied the genetic basis of subclinical brain disease, both in genome-wide settings and in candidate-gene studies [296–302] (see further section on population imaging).

Most research on the preclinical phase of neurodegenerative diseases, particularly dementia, focuses on either cognitive performance or brain pathology on neuroimaging. Given that brain pathology is usually diffuse, it is conceivable that brain functions other than cognition will also be affected. In this light, we have shown that gait deteriorates significantly with age [303]. Also, gait and cognition are linked following a specific pattern of certain cognitive domains only associated with specific aspects of gait, but not others [303]. Recently, we have reported our first findings with migraine and cerebral hemodynamis [304].

### Methods update

#### Assessment of dementia and Alzheimer disease

In the baseline and follow-up examinations participants undergo an initial screen for dementia with the Mini Mental State Examination (MMSE) and the Geriatric Mental Schedule (GMS), followed by an examination and informant interview with the Cambridge Examination for Mental Disorders of the Elderly (CAMDEX) in screenpositives (MMSE < 26 or GMS > 0), and subsequent neurological, neuropsychological and neuroimaging examinations [251, 253]. Of subjects who cannot be reexamined in person, information is obtained from the GPs and the regional institute for outpatient mental health care. A consensus panel makes the final diagnoses in accordance with standard criteria (DSM-III-R criteria; NINCDS-ADRDA; NINDS-AIREN).

#### Assessment of Parkinsonism and Parkinson disease

Participants are screened in the baseline and follow-up examinations for cardinal signs of parkinsonism (resting tremor, rigidity, bradykinesia, or impaired postural reflexes). Persons with at least one sign present are examined with the Unified Parkinson’s Disease Rating Scale and a further neurologic exam. PD is diagnosed if two or more cardinal signs are present in a subject not taking antiparkinsonian drugs, or if at least one sign has improved through medication, and when all causes of secondary parkinsonism (dementia, use of neuroleptics, cerebrovascular disease, multiple system atrophy, or progressive supranuclear palsy) can be excluded [252].

#### Assessment of stroke and stroke subtypes

History of stroke at baseline was assessed through interview and verified in medical records. Putative incident strokes get identified through the linkage of the study database with files from general practitioners, the municipality, and nursing home physicians’ files, after which additional information (including brain imaging) is collected from hospital records. A panel discusses all potential strokes and subclassifies strokes into ischemic, hemorrhagic or unspecified [254, 271]. We also systematically collect transient ischemic and neurological attacks [305].

#### Assessment of cognitive function

Global cognitive function is measured through the Mini Mental State Examination (MMSE) in all surveys. From the third survey (RS-I-3) onwards we added a 30 min test battery that was designed to assess executive function and memory function, and which includes a Stroop test, a Letter Digit Substitution Task, a Word Fluency Test, and a 15 words Word List Learning test. This test battery was expanded from the fourth survey onwards (RS-I-4) to include motor function assessment using the Purdue Pegboard Test. Moreover, from 2009 onwards we expanded further by including the Design Orientation Test (DOT) and a modified version of the International Cooperative Ataxia Rating Scale (ICARS), which assess visuo-spatial orientation and ataxia respectively [306–308].

#### Assessment of gait patterns

Halfway through RS-III-1, we successfully implemented the assessment of gait in all participants using the GAITRite walkway (http://www.gaitrite.com/). Gait is assessed using a 5.79 m long walkway (GAITRite Platinum; CIR systems, Sparta, NJ, USA: 4.88 m active area; 120 H sampling rate) with pressure sensors. Participants perform a standardized gait protocol consisting of three different walking conditions: normal walk, turning and tandem walk. In the normal walk, participants walk over the walkway at their own pace. This walk is repeated four times in both directions (yielding a total of 8 recordings). In turning, participants walk over the walkway at their own pace, turn halfway and return to the starting position (1 recording). In the tandem walk, participants walk tandem (heel-to-toe) over a line visible on the walkway (1 recording). A total of 30 spatiotemporal gait variables are calculated by the walkway software and downloaded offline for further analysis. Subsequently, principal components analysis on these thirty gait variables is performed to derive summarizing factors, referred to as gait domains. The following gait domains are used: Rhythm, Pace, Phases, Base of Support, Variability, Tandem, and Turn. Gait domains can be compared to cognitive domains, in which each domain reflects a different aspect of the overall concept [303].

#### Assessment of polyneuropathy

Starting in January 2013, we have successfully implemented a protocol to assess polyneuropathy. This includes a full work-up including questionnaire, neurological exam, and EMG in all participants. In coming years, we will publish on the prevalence, risk factors, and clinical correlates of polyneuropathy in the general population. The continuous measures of conductivity obtained through EMG can also serve as excellent endophenotype for genetic and biomarker studies.

#### Rotterdam Scan Study: brain imaging within the Rotterdam Study

In 1991, a random sample of 111 participants underwent axial T2-weighted magnetic resonance (MR) imaging to assess presence and severity of white matter lesions [284]. In 1995, a random sample of 563 non-demented participants underwent brain MR imaging in the context of the Rotterdam Scan Study. From August 2005 onwards (RS-II-2 and further), a dedicated 1.5 T scanner is operational in the research center of the Rotterdam Study, and brain imaging is performed in all study participants without contra-indications [285].

Currently, the follow-up of this latter sample extends to up to 10 years (see further section on population imaging).

For additional EJE references see [99, 159, 264, 309–329].

## Ophthalmic diseases

### Objectives

Ophthalmic research in the Rotterdam Study focusses on occurrence, causally related determinants, and predictors of common eye diseases. Our main focus is on age-related macular degeneration (AMD), glaucoma and myopia, but we study retinal vessel diameters and (diabetic) retinopathy as well. In the previous studies, we mostly focused on common genetic risk variants derived from GWAS and environmental factors. We will now move on to the study of rare genetic variants, genetic effects on phenotype, in silico pathway analysis, biomarkers, and prediction models.

### Major findings

#### Age-related macular degeneration (AMD)

AMD is one of the few complex traits of which a large proportion of the genetic background has been revealed. Currently known are 45 loci which show association with common variants, and a handful of genes which harbor rare genetic variants. Four genes have very common SNPs with major effects, and carriers of more than one of these variants have a more than 50 % chance of developing AMD. Many of the identified genes play a role in the lipid pathway. Together with the 3 continent AMD consortium [330], we studied lipid genes in conjunction with serum lipid levels and incidence and progression of AMD in a total of 6950 participants. After adjustment for all confounders, we found no significant relationship between lipid levels, lipid genes, or statin-use in this meta-analysis. This points out that potential relationships between cardiovascular determinants and AMD may be indirect.

As hormones have been hypothesized to play a role in AMD, we studied thyroid-stimulating hormone (TSH) and free thyroxine (FT4) [202]. 5573 participants from the Rotterdam Study were followed for an average period of 6.9 years during which 805 persons developed AMD. Higher FT4 levels were associated with a higher risk of AMD, and participants in the highest quintile had a 1.3 fold increased risk of developing AMD. This relation was also found in euthyroid participants, indicating that this relationship exists already at a subclinical level.

Our gene-environment analyses focused on nutrition. We pooled the dietary data that we collected at baseline with data from the Australian population-based Blue Mountain Eye Study, and evaluated development of AMD in 4632 persons during 15 years of follow up [331]. For those at high genetic risk (based on carriership of the CFH and ARMS2 genes), we found a strongest risk reduction when their intake of lutein/zeaxanthin (risk reduction 20 %) and fish (risk reduction 40 %) was high. These data are highly clinically relevant, as they provide leads for doctors on how to advise patients at high genetic risk to alter their fate.

Direct-to-consumer companies have exploited the recent genetic advances to market genetic testing for AMD. We aimed to evaluate the merit of these companies by sending DNA samples of our own researchers to these labs, as well as to our own Rotterdam Study laboratory using our own developed prediction model [332]. The results from the companies showed a 1.6-fold difference for overall relative risk to an up to 12-fold difference for lifetime risk. Most important reasons for the differences in risks were the testing of only a limited set of genetic markers, the choice of the reference population, and the methodology applied for risk calculation. We believe there is ample room for improvement here.

#### Myopia (nearsightedness)

A myopia boom is taking place all over the world. We and others found an increasing prevalence of myopia as well as a rise of the more extreme end of the spectrum, high myopia (refractive error of -6 diopters and beyond). In collaboration with studies in the European Eye Epidemiology consortium (E3), we performed a meta-analysis of refractive error prevalence [333]. We used the 2010 European Standard Population and studies ages between 25 and 90 years. The prevalence standardized to age 50 in Europe were 30.6 % myopia, 2.7 % high myopia, 25.2 % hyperopia and 23.9 % astigmatism. However, the prevalence of myopia was up to 47 % in those aged 25–29 years, showing a generation increase. In the search for explanations for this strong rise, we explored the association with education [334]. We found that higher education is strongly associated with myopia, but it seems to be an additive effect and does not explain the entire frequency rise.

The clinical relevance of myopia is determined by its visual consequences. We evaluated the risk of severe visual impairment for the entire range of refractive errors, and found that high hyperopia as well as high myopia carried an increased risk of visual impairment [335]. High myopia had the most severe visual consequences. One in three high myopes developed severe visual impairment during his/her lifetime, and risks went up to >90 % for those with axial lengths of 30 mm and higher. The most common cause of visual impairment was irreversible myopic macular degeneration.

In recent years, we acquired more knowledge on the genetic background of myopia. With our international CREAM consortium (Consortium of Refractive Error And Myopia), we further explored common genetic associations with axial length (AL). We replicated the association with the ZC3H11B gene [336], and found eight other genome-wide significant loci in a meta-analysis. These loci were in linkage disequilibrium with the genes RSPO1, C3orf26, MIP, ZNRF3, LAMA2, GJD2, CD55, ALPPL2 and ZC3H11B. The last five were also associated with refraction, indicating significant overlap between AL and refractive error. In addition to AL, we focused on the extreme ends of spherical refraction, and found 11 loci to be associated with both myopia and hyperopia [337]. The effects of the risk alleles in these loci were in opposite directions, providing support for the existence of only one refractive error trait. We also focused on astigmatism, a refractive error which results from radial inconsistencies of the cornea as well as the lens [338]. Astigmatism is a common companion of myopia and hyperopia, and is thought to have a genetic basis as well. We conducted a meta-analysis on refractive astigmatism in CREAM, and found a significant hit with a marker downstream of the neurexin-1 (NRXN-1) gene (P = 3.92E−8). No other genome wide significant loci were found. This suggests a more complex genetic background for astigmatism than for spherical aberrations. We will continue to pursue the search for astigmatism genes the coming years.

Future research on myopia will be focused on finding the missing genes with exome analyses, whole genome analyses, and family studies; on pathway analysis and functional studies; and on development of prediction models.

#### Primary open-angle glaucoma (POAG)

Although many genetic risk factors for primary open-angle glaucoma (POAG) and POAG related endophenotypes have been identified these last years, a large part of the heritability is still unexplained. To this end, we combined our efforts with international study groups in the International Glaucoma Genetics Consortium (IGGC). This consortium investigated GWAS data on POAG and POAG-related endophenotypes including intraocular pressure (IOP), vertical cup-disc ratio (VCDR), cup area (CA) and disc area (DA).

### Intraocular pressure

A meta-analysis focused on GWAS of IOP was performed including 35,296 subjects from 7 countries participating in IGGC [339]. Four new IOP-associated loci were identified (FNDC3B, ABCA1, ABO, and a region on chromosome 11p11.2). Three genes also showed significant association with POAG. In a GWAS study of IOP in the Rotterdam Study I and Rotterdam Study II using 1000 Genomes imputations, we identified a new locus (ARHGEF12) for IOP [340]. We replicated this finding in 5 other population-based studies, and found a significant association with POAG in two independent case–control studies. The ARHGEF12 gene is involved in IOP regulation via the RhoA/RhoA kinase, and novel drugs targeting this pathway show promising results.

### Vertical cup–disc ratio

A meta-analysis of VCDR was performed in 27,878 individuals [341]. Ten novel loci associated with elongation of the cup were identified (COL8A1, DUSP1, EXOC2, PLCE1, ADAMTS8, RPAP3, SALL1, BMP2, HSF2 and CARD10). Persons in the highest quintile of genetic risk had a 2.5-fold increased risk of POAG compared with those in the lowest quintile. Pathway analysis implicated a negative regulation of cell growth and cellular response to environmental stress as key pathological pathways.

### Optic disc area parameters

A meta-analysis investigating CA and DA in 24,089 individuals identified 10 novel loci for CA (DHRS3, TRIB2, EFEMP1, FLNB, FAM101, DDHD1, ASB7, KPNB1, BCAS3, and TRIOBP) and 10 novel loci for DA (CDC42BPA, F5, DIRC3, RARB, ABI3BP, DCAF4L2, ELP4, TMTC2, NR2F2, and HORMAD2) [342]. These loci explained an additional part of the variance in glaucoma. These results showed that CA and DA—measurements were of additional value in the identification of causal factors for glaucoma.

### Functional genetics of POAG

Our previous glaucoma studies had showed that the SIX1/SIX6 locus was associated with VCDR and POAG. Fine mapping and exome sequencing of this region revealed 2 new variants in the SIX6 gene and pointed to SIX6 as the responsible gene for the associated signal. We further aimed to investigate the functional consequences of this gene by studying the effect of a transient knockdown of SIX6 using morpholinos [343]. We found that absent function of SIX6 led to a 30–50 % decrease in eye dimensions. Histologic examination revealed an underdeveloped lens and a thin optic nerve. Expression analysis revealed up-regulation of CDKN2B, a cyclin-dependent kinase inhibitor that is involved in cell cycle regulation and TGFß-induced growth inhibition that has been consistently implicated in POAG. These results confirm that SIX6 plays a role in eye growth and development, and that regulation of the CDKN2B protein appears to be a downstream mechanism. Future steps for POAG genetics will focus on 1000 Genomes imputations, and analysis of exome chip and exome sequencing data.

### POAG diagnostics and imaging

All over the world, retinal imaging is becoming more and more a diagnostic tool for POAG. Imaging with optical coherence tomography (OCT) has made it feasible to visualize and measure the retinal ganglion cell and nerve fiber layer, two structures which are key elements in glaucoma. We collaborated with the Iowa Imaging group led by Michael Abramoff to measure the various layers of the retina using automated digital algorithms. Together with the Iowa group, we determined the screening performance of OCT layer thickness measurements in the peripapillary and macular region for POAG in the Rotterdam Study [344]. Mean thickness of the retinal ganglion cell layer in the inferior half of the macular region showed the highest predictive value (AUC 0.85; 95 % CI 0.77–0.92) and sensitivity (53.7 %; 95 % CI 38.7–68.0 %) for glaucoma. The mean thickness of the peripapillary retinal nerve fiber layer had AUC 0.77 (95 % CI 0.69–0.85) and sensitivity 24.4 % (95 % CI 13.7–39.5 %). These data shed new light on the occurrence of glaucomatous events at the cellular level, and may indicate that the macula is more important as an initiation site than the optic disc.

### Methods update

At baseline and follow-up examinations participants undergo ophthalmic measurements including best-corrected ETDRS visual acuity, refractive error, Goldmann applanation tonometry, keratometry, slit lamp examination of the anterior segment and visual field testing. In pharmacological mydriasis we make 35 color photographs of the macular area, and 20 simultaneous stereoscopic imaging of the optic disc and macular area. Since the fourth follow-up, 35 stereoscopic color photographs of the optical disc and the macular area were made (RS-I-5). Analog fundus photography was replaced by stereoscopic digital imaging of the macular area and optic disc since the third follow-up examination. Optic nerve head analysis with a Heidelberg Retina Tomograph, macular pigment density, and melanin optical density measurements were added during the third follow-up (RS-I-3). At fourth follow-up examination, fourier domain optical coherence tomography of the macular area and optical disc, axial length and width measurements of cornea, anterior chamber, lens, posterior chamber and retina measured with Lenstar; and fundus autofluorescence, infra-red and red-free measurements were added (RS-I-5).

The classification of AMD, POAG, refractive error, and retinal vessel diameters remained unchanged.

For additional EJE references see [345–349].

## Psychiatric diseases

### Objectives

The aim of the psychiatric research in the Rotterdam Study is to investigate the determinants, correlates and consequences of common psychiatric problems in the elderly. The focus lies on studies of depressive and anxiety disorders, sleep disturbances, addiction to smoking, and complicated grief.

### Study design update

Initially, the psychiatric data collection was very limited but this was expanded strongly in the last 15 years. In the second visit most participants were screened for depressive symptoms and from the third examination onwards (RS-I-3), in 1997–1999, depressive disorders have been ascertained systematically. Assessments of anxiety disorders, sleeping disturbances, and complicated grief were added in the subsequent examination (RS-I-4) and have been performed in all follow-up visits of the original and added cohorts. Recent additions to the protocol included a screening for psychotic symptoms and, from January 2012–October 2014, ambulatory polysomnography.

### Major determinants

Psychiatric research in the Rotterdam Study focuses on biological risk factors. The vascular depression hypothesis was tested with different measures of atherosclerosis, arterial stiffness and cerebral blood flow [350]. We examined whether blood levels of vitamins and fatty acids, immune parameters, and markers of folate metabolism increased the likelihood of depression. Diurnal patterns of cortisol secretion were related to psychiatric and other outcomes such as subclinical atherosclerosis. Within the psychiatric research line, very few candidate gene studies were performed, whereas several GWAs were conducted in collaborative efforts focusing on depressive symptoms, sleep, anxiety and cortisol [351–354]. Several, mostly cross-sectional studies of brain morphology as possible determinants and correlates of common psychiatric disorders were completed [292]. Current data collection includes a dexamethasone suppression test to measure hypothalamic–pituitary–adrenal axis activity in all participants, which is unique in a population-based study. Also, psychiatric problems and psychological traits such as happiness, sleep duration, and depression are increasingly studied as determinants of health and mortality [355].

### Methods update

#### Assessment of depressive disorders and symptoms

Information on depression is obtained from (a) psychiatric examinations, (b) self-reported histories of depression, (c) medical records, and (d) registration of antidepressant use [356]. The psychiatric examination during each visit consists of a assessment and screening with the Center for Epidemiologic Studies Depression Scale (CES-D).All screen-positive participants identified by a CESD score of 16 or above in each follow-up examination, are interviewed by a clinician (psychiatrist, psycho-geriatrician or clinical psychologist) with the semi-structured clinical interview (Dutch version of the Schedules for Clinical Assessment in Neuropsychiatry-SCAN) to diagnose depressive disorders. Major depressive disorders are classified according to the Statistical Manual of Mental Disorders, 4th revised edition (DSM-IV) criteria. To continuously monitor incidence of depression throughout follow-up, information on the occurrence of episodes of depression and depressive symptoms are continuously collected from general practitioners medical records. All medical records such as hospital discharge letters, specialists reports, and notes of the GP are extracted and copied by a research-assistants looking for potential depressive symptoms. These extracted data are rated and categorized by two medical doctors. Consensus decisions are made for disagreeing categorizations. Finally, incident episodes of MDD were defined as the first event that chronologically occurred in one of the two data sources described above.

#### Assessment of anxiety disorders

Anxiety disorders are diagnosed as part of the home interview. Trained lay interviewers conduct a slightly adapted version of the Munich version of the Composite International Diagnostic Interview (M-CIDI) to assess the following anxiety disorders with a computerized diagnostic algorithm according to DSM-IV criteria: generalized anxiety disorder (GAD), panic disorder, agoraphobia, social phobia and specific phobia [357]. The M-CIDI was specifically designed to obtain DSM-IV diagnoses and test–retest reliability for anxiety disorders is good (kappa for any anxiety disorder: 0.81). For all anxiety disorders, except GAD, one-year prevalence is assessed. In addition, the HADS-A is used to assess anxiety traits continuously.

#### Assessment of sleep and circadian rhythms

Sleep quality and disturbance is measured with the Pittsburgh Sleep Quality Index. In addition, sleep duration and fragmentation are assessed with actigraphy, a method that infers wakefulness and sleep from the presence or absence of limb movement [358]. In total, nearly 2000 persons participated in this actigraphy study: they wore an actigraph and kept a sleep diary for, on average, six consecutive nights. Follow-up assessments of actigraphic assessments in these participants are currently scored. Ambulatory polysomnographic (PSG, i.e., full sleep EEG) recordings of one night have been conducted in 940 participants. We scheduled home visits of a research assistant who placed the sensors to record an ambulant PSG (Vitaport 4; Temec, Kerkrade, the Netherlands). The PSG included six EEG channels, F3/A2, F4/A1, C3/A2, C4/A1, O1/A2, O2/A1, bilateral electrooculography, electromyography, electrocardiography, respiratory belts on the chest and abdomen, oximetry, and a nasal pressure transducer and oronasal thermocouple to measure airflow [359]. All recordings were scored according to American Association of Sleep Medicine guidelines by a registered Sleep Technologist. Recordings were manually scored in 30-s epochs for identification of sleep stages; each epoch was scored as Wake, N1, N2, N3 or REM sleep. For each sleep stage, the duration and latency was determined. In addition, we used PRANA (PhiTools, Strasbourg, France) software to automatically measure the microstructure of sleep, e.g. spindles and REM density. Polysomnography recordings are also used to calculate the apnea hypopnea index.

Circadian rhythms: Sleep-wake activity patterns over a week are studied with actigraphy as a marker of circadian rhythms. In more than 1700 persons we calculated interdaily stability, i.e. the stability of the rhythm over days and the intra-daily variability, i.e. the fragmentation of the rhythm [360].

#### Assessment of grief

All participants are asked if they are currently grieving. If the answer is positive we ask formal follow-up questions “When did this person die?”, and “Who was this person?” The participants who answered the first question affirmatively are assessed for complicated grief with the Dutch version of the Inventory of Complicated Grief (ICG). The ICG is considered the gold standard for measurement of complicated grief in older adults because it has high internal consistency, good convergent and criterion validity.

### Major findings

Depression: The incidence and recurrence of depression in the elderly was estimated by continuously monitoring depression during a follow-up period of, on average, 8 years [356]. In total, 566 depressive syndromes and 1073 episodes of clinically relevant depressive symptoms occurred. For depressive syndromes, the incidence rate was 7.0 (95 % CI 6.0–8.3) per 1000 person-years and the recurrence rate was 27.5 (95 % CI 23.7–32.1) per 1000 person-years. The recurrence rate of depressive syndromes was equal for women and men.

In a series of studies we found some evidence for the vascular depression hypothesis. More severe coronary and extra-coronary atherosclerosis were associated with a higher prevalence of depression, as were cerebral haemodynamic changes [350]. However, our data did not support a specific symptom profile of vascular depression as previously defined. Most importantly, we found no longitudinal relation between peripheral atherosclerosis and incident depression [361]. Recently, we prospectively studied cerebral vascular risk factors such as white matter lesions, silent infarcts or blood flow in relation to depression [362]. We found evidence that small vessel disease predicted the onset of depression. This suggests that atherosclerotic processes in the brain are a specific risk factor for depression.

Sleep: We investigated the relationships of sleep duration with both cardiovascular risk factors and psychiatric disorders. We also aimed to explain sex differences in subjective and actigraphic sleep parameters [363]. If assessed by diary or interview, elderly women consistently reported shorter and poorer sleep than elderly men. In contrast, actigraphic sleep measures showed shorter and poorer sleep in men. These discrepancies were partly explained by sleep medication use and alcohol consumption. The first results using polysomnography to measure sleep EEG suggest that REM-density is a marker of depressive symptoms in the general population [359]. Other results suggest that sleep apnea and depressive symptoms are not related, although both result in fatigue [364].

Anxiety: We found that prevalent anxiety disorders fulfilling DSM-IV criteria may be much less co-morbid with depressive disorders than previously thought if the disorders are assessed with different diagnostic instruments. On the other hand, a history of depression is very common in persons with prevalent anxiety disorder [365].

Complicated grief: In our population-based study of 5741 elderly persons, current grief was reported by 1089 participants, of these 277 (25 or 4.8 % of total) were diagnosed with complicated grief, the vast majority of which had no clinical symptoms of anxiety or depression. Persons with complicated grief were older, had a lower level of education, and more often had lost a child [366]. Recently published work suggests that complicated grief occurs together with structural brain atrophy more often than expected by chance [367].

Genetics of common psychiatric disorders: In the past years, we have performed a series of genome-wide association studies of the above psychiatric and psychological phenotypes, mostly as part of the CHARGE consortium. Initial analyses have yielded no convincing genome wide significant results as studies were strongly underpowered, psychiatric phenotypes do not present very homogenous entities and are highly polygenetic. Disappointingly, the genome wide analyses of intermediate phenotype in the field of psychiatry such as cortisol or executive function have hardly been more successful [368, 369]. However, more recent work with larger sample sizes as part of the PGC consortium shows promising results.

Finally, ongoing psychiatric research projects examine whether and how psychological well-being or psychiatric problems contribute to survival. Most importantly, we are interested in whether the effects are specific to certain behaviour or emotions, are independent of confounding by physical disease, or can be explained by lifestyle, immunological or hormonal regulation [370].

For additional EJE references see [153, 371–384].

## Respiratory diseases

Within the Rotterdam Study, the prevalence, incidence and natural history of common respiratory diseases in older subjects are investigated. The main focus of the respiratory epidemiology group is chronic obstructive pulmonary disease (COPD), but the research group also investigates acute respiratory tract infections, pneumonia, asthma, asthma and COPD overlap syndrome (ACOS) and lung cancer. In addition, the respiratory epidemiology group aims to elucidate the clinical and genetic determinants of lung function, encompassing spirometry and diffusing capacity of the lungs. Several complementary epidemiologic approaches are performed within the Rotterdam Study: clinical epidemiology, genetic epidemiology, molecular epidemiology and pharmaco-epidemiology. Thanks to an excellent collaboration with the investigators of the Generation R birth cohort study, also life course epidemiologic studies are performed to elucidate the early life origin of complex respiratory traits and diseases in adulthood [385].

### Lung function

At each cross-sectional round of paraclinical investigations at the Ommoord research center, spirometry is performed using a Master Screen^®^ PFT Pro by trained paramedical personnel according to the American Thoracic Society (ATS)/European Respiratory Society (ERS) guidelines [386.] In the most recent round of paraclinical investigations (from March 2009 till June 2014), diffusing capacity of the lungs (DLCO) has been measured in all participants of the three cohorts (RS I-5, RS II-3 and RS III-2) using the Master Screen^®^ PFT Pro according to the ATS/ERS guideline on standardisation of the single-breath determination of carbon monoxide uptake in the lung [387]. The aim of the measurements of spirometry and diffusing capacity of the lungs is fourfold: first, to diagnose COPD according to the Global initiative for Obstructive Lung diseases (GOLD) guidelines; second, to determine the severity of disease as evidenced by the severity of airflow limitation in those subjects with an obstructive spirometry (defined as a ratio of Forced Expiratory Volume in one second (FEV1) to Forced Vital Capacity (FVC) <70 %); third, to phenotype COPD subjects accurately by taking into account emphysema as evidenced by a decreased DLCO; and finally, to investigate the decline of lung function over time as a marker of disease activity and progression in COPD.

In collaboration with the CHARGE consortium, we have performed landmark Genome Wide Association Studies (GWAS) of lung function, which clearly implicate early life origins of adult lung function. The first GWAS of the FEV1/FVC ratio, a spirometric measure of airflow limitation (mainly due to airway obstruction), revealed eight genetic loci [388]. Interestingly, using the hypothesis-free GWAS approach, two genetic loci—near the hedgehog interacting protein (HHIP) gene on chromosome 4 and in the gene patched 1, the receptor for hedgehog, on chromosome 9—were significantly associated with airflow limitation, and appeared to be involved in the same biological pathway, namely the hedgehog pathway. This hedgehog pathway regulates the delicate intercellular communication between lung epithelial cells (derived from the endoderm) and lung mesenchymal cells such as smooth muscle cells (derived from the mesoderm) in foetal life, and is crucial for the branching morphogenesis of the lungs. In the second GWAS of FEV1/FVC in more than 90,000 subjects, thanks to a collaboration between the CHARGE and Spirometa consortia, 16 additional genetic loci have been discovered, including genes involved in oxidative stress responses (GSTD), homeostasis of extracellular matrix proteins (ADAM19, MMP15), Transforming Growth Factor-β (TGFB) signalling (TGFB2) and branching morphogenesis (RARB) [389]. Together, these two GWAS implicate that early life events during embryonic life and infancy might contribute to lung function in adulthood. Recently, we—in collaboration with the CHARGE consortium—have published a GWAS of Force Vital Capacity (FVC), a marker of lung volume and an independent predictor of survival in adult populations (hence the word “vita” or “life” in the term FVC) [390].

### Chronic obstructive pulmonary disease

The prospective population-based Rotterdam Study is ideally suited to investigate key research questions of Chronic Obstructive Pulmonary Disease (COPD) regarding its pathogenesis, epidemiology, environmental and genetic risk factors, natural history of the disease, phenotypes, exacerbations and co-morbidities [391]. Based upon results of spirometry, medical files of general practitioners and letters of respiratory physicians, we have currently a validated diagnosis of COPD in approximately 2000 participants (out of the total cohort of 15,000 subjects), with a median follow-up of 12 years, implicating more than 24,000 person-years of follow-up [392]. Importantly, the COPD participants are well phenotyped, thanks to a combination of crucial informations: (1) questionnaire-based data (e.g. respiratory symptoms including shortness of breath, cough and sputum), (2) smoking history, (3) medical information on chronic diseases and acute events (based upon medical files from general practitioners and hospital letters), (4) lung function tests (spirometry and diffusing capacity of the lung), (5) exacerbation history (of both moderate exacerbations and severe exacerbations requiring hospitalisation), (6) drug dispensing data (of oral and inhaled treatments) and (7) mortality data in nearly all COPD subjects. In addition, in a large random sample of Rotterdam Study participants (n = 2500), CT scans of the chest have been performed for imaging of the heart, coronary arteries and mediastinal large vessels, but also providing imaging of the lungs at inspiration and the opportunity to quantify the degree and extent of emphysema.

COPD does not only affect the airways and lungs, but is also associated with extra-pulmonary manifestations and co-morbidities. In the Rotterdam Study, we have demonstrated that COPD is associated with cerebrovascular macro- and micro-angiopathy [393, 394]. In a cross-sectional analysis, COPD cases had a twofold increased prevalence of carotid artery wall thickening on ultrasonography compared with controls with normal lung function [393]. Importantly, the risk of carotid wall thickening increased significantly with severity of airflow limitation. Moreover, on magnetic resonance imaging (MRI) of the extracranial carotid arteries, lipid core plaques were more frequent in COPD subjects than in control subjects. Since lipid core plaques are more vulnerable to rupture, this might predispose COPD patients to the occurrence of stroke (especially ischemic stroke). In a second study on cerebrovascular co-morbidities in COPD, we demonstrated that subjects with COPD had a higher prevalence of cerebral microbleeds on MRI of the brain, which is a novel marker of cerebral small vessel disease [394]. The association was independent of age, sex, smoking status, atherosclerotic macroangiopathy, serum creatinin, total cholesterol and triglycerides. Intriguingly, regarding the specific location of the cerebral microbleeds, COPD subjects had a significantly increased prevalence of microbleeds in deep or infratentorial locations. These findings were confirmed in longitudinal analyses restricted to subjects without cerebral microbleeds at baseline, since COPD was an independent predictor of incident cerebral microbleeds in deep or intratentorial locations [394]. To what extent these imaging abnormalities contribute to the observed gait disturbances in patients with COPD [395], needs to be elucidated.

Several studies have shown that COPD is associated with cardiovascular diseases, encompassing ischemic heart disease, acute myocardial infarction, cardiac dysrhythmia (e.g. atrial fibrillation) and heart failure. Recently we have demonstrated that subjects with COPD have an increased risk of sudden cardiac death [396]. The risk increased particularly 5 years after diagnosis of COPD and in patients with frequent exacerbations of COPD. Since in observational studies in patients with COPD beta-blockers have been shown to reduce mortality, and since we have demonstrated that the effects of cardio-selective beta-blockers on pulmonary function are minimal in the general population and in COPD subjects [397], it is time to perform interventional studies with beta-blockers in patients with COPD in order to reduce the high cardiovascular and all-cause mortality in this vulnerable patient population.

Since COPD is characterized by chronic pulmonary inflammation and an accelerated ageing of the lung, we have investigated whether COPD is associated with frailty. Using the Fried criteria of (physical) frailty, evaluating nutritional status (unintentional weight loss), grip strength, mobility (slow walking speed), physical activity and exhaustion, we have determined the prevalence of frailty in 2.833 participants of the Rotterdam Study (median age: 74 years): 5.8 % were frail [229]. Frail elderly had lower quality of life, had more frequently fallen or been hospitalized, and were at increased risk of dying within three years compared to non-frail elderly [229]. The prevalence of frailty in subjects with COPD (10.2 %) was significantly higher than in subjects without COPD (3.4 %). After adjusting for age, sex, smoking, corticosteroids and other confounders, COPD subjects had a more than twofold increased prevalence of frailty [395]. Importantly, COPD elderly who were frail had a significantly worse survival.

Additional EJE references [398–403].

## Genomics and biomarker studies

### Objectives

The team in this research line focusses on bio-banking activities of the participants of the Rotterdam Study and investigates biological determinants of disease (i.e., DNA, RNA, proteins, metabolites, microbes, etc.). Bio-banking involves collecting, storing and managing the biological tissues of participants of the Rotterdam Study at all follow-up measurements. This concerns mainly blood, urine, saliva, hair and faeces. We have further started to store full blood samples for the isolation of induced pluripotent stem cells. The research focus of this group concerns assessment of biological determinants of disease (biomarkers) in these biomaterials and the analysis of markers using genomic technologies (such as SNP arrays and next generation sequencing (NGS)).

### Major findings

Rotterdam Study investigators are playing leading roles in several of the large global consortia focused on assessing the contribution of complex disease gene variants by prospective meta-analysis across many epidemiological cohorts, such as in CHARGE and ENGAGE, and in many disease/phenotype focused efforts such as ADSP, IGAP, PERADES, GIANT, GEFOS, REPROGEN, TREATOA, DIAGRAM, etc. Since 2005 the genome wide association study (GWAS) has changed the field of complex genetics, and identified a still growing list of common variants contributing to disease risk and explaining genetic variance of traits. While this large scale global collaboration has originated from the GWAS era, we now see similar consortia being built around the newer genomics datasets with RNA expression profiles, DNA methylation profiles, and the NGS datasets on DNA, RNA and microbiomes, including the BBMRI-NL sponsored BIOS consortium.

The Rotterdam Study has GWAS data for almost the complete dataset summing to over 12,000 DNA samples, and is involved as a major collaborative centre for meta-analysis studies of GWAS data, including national programs (RIDE, NGI-NCHA), EU-funded projects (GEFOS, TREATOA, ENGAGE), and voluntary collaborations (GIANT, MAGIC, CHARGE). Especially, from the CHARGE consortium (the Rotterdam Study together with the Framingham Study, AGES, CHS, and ARIC) many important publications have emerged on a wide variety of phenotypes and diseases from all major research lines in the Rotterdam Study [69]. You can find them discussed under the subheadings of each individual research line.

### Data collection, storage and management

At each examination at the research centre, blood, serum, plasma (citrate, heparine), and saliva is collected, as well as EDTA tubes for DNA and PAXgene tubes for RNA isolation. Fasting blood samples are collected along with challenged samples as part of a glucose tolerance test. Saliva is collected before and after a dexamethasone-suppression test. Saliva is frozen at −196 °C before and after the challenge and stored at −80 °C. To obtain serum and plasma, tubes are centrifuged according to a protocol standardising time and conditions from the drawing of blood to centrifugation. All samples including the full blood are snap frozen at −196 °C using liquid nitrogen and stored at −80 °C. Overnight urine samples are collected at home, frozen at −196 °C at the research centre and stored at −80 °C.

DNA is isolated from whole blood at one laboratory at Erasmus MC by a manual salting-out protocol and is subsequently stored in Eppendorf tubes at −20 °C. A copy of the complete DNA collection of ~13,000 samples has been transferred to Matrix 2D-barcode tubes in 96-well format at another location. This copy has been subjected to normalization of DNA concentrations and made suitable for handling in 96- and 384-well micro-titer plates for subsequent downstream genomic analysis.

Starting with the RS-III round of data collection, blood drawing has also been taken place with PAXgene tubes, from which whole RNA is isolated and stored at −80 °C. This is now ongoing for the whole study population following the cycles of visits to the research centre.

Similarly, with the RS-III round, collection of faeces material has been initiated for the intestinal microbiome analysis. A collection pot is distributed at the research centre visit which is to be used at home and then by postal mailed returned to Erasmus MC where DNA is isolated and stored at −80 °C. This is now ongoing for the whole study population following the cycles of visits to the research centre.

For data management, an in-house customized sample-management system has been developed. All genomic data of the Rotterdam Study (e.g., SNP array, RNA expression, NGS DNA and RNA, microbiome 16S NGS) are generated in one laboratory which keeps all raw data, while QC-ed and extracted data are stored and managed in the central data repository of the Rotterdam Study.

### Blood serum/plasma assessments

For all participants, serum cholesterol, HDL, LDL, triglycerides, glucose and glucose levels are assessed. In urine, micro albumin and creatinine are determined in all participants. Recently, a new “baseline” serum biomarker dataset has been generated at the Erasmus MC Clinical Chemistry Laboratory and the Endocrine Laboratory consisting of RS-I-3, RS-II-2, and RS-III-1 samples (N = ~10,000). These measurements include a steroid profile by mass-spectrometry (e.g., estrogens, androgens, cortisol), vitamin D, thyroid hormones (TSH, free T4), interleukins, CRP, IGF1, insulin, iron-parameters (iron, ferritin and transferrin saturation), fibrinogen, homocysteine, folic acid, riboflavine, pyridoxine, SAM/SAH ratio, cobalamine, Lp-PLA2, Fas/Fas-L, and abeta42/40.

### Human genomics facilities

The Rotterdam Study uses one Erasmus MC laboratory (the Human Genotyping Facility, HuGE-F, at the department of Internal Medicine) for all its genomic studies on DNA, RNA, methylation, microbiome, etc. The facility use high-end automated machinery including 2 Caliper pipetting robots (including a TwisterII, and integrated plate reader (OD 260/280), 2 Tecan EVO 150 Freedom pipetting robots, a Deerac Equator NS808 nanoliter liquid dispenser, PCR machines, an ABI7900HT Taqman machine (running 1 ng gDNA in 2 microliter reactions), 2 Illumina iScan micro-array readers, one Roche 450 GS Junior sequencer, two Illumina HiSeq2000 sequencing machines, and has access to Illumian HiSeq2500 and Myseq machines. DNA sample handling is centred on 384-well plates. Single SNP genotyping studies are done mostly using Taqman and Sequenom genotyping with throughputs at 30,000 genotypes per day. We work with reduced amounts of input genomic DNA of 1 nanogram per genotype. This facility has been generating all GWAS data for the Rotterdam Study as well as its RNA expression profiles, methylation profiles, and all NGS data including whole exome sequences, RNA sequencing, and microbiome 16S sequencing.

### Genome-wide association studies (GWAS) datasets

GWAS are based on genotyping epidemiological cohorts with high density SNP arrays with 500,000 - 5 million SNPs. The method has been shown to successfully identify common genetic factors for hundreds of traits and diseases (see www.genome.gov/GWAstudies). Through a large grant from the Dutch research organisation NWO in 2007 one of the world’s largest GWAs datasets has been facilitated involving over 12,000 DNA samples from the Rotterdam Study cohorts. This GWAS dataset consists of a) a small dataset of ~450 women with 500 K Affymetrix arrays (Nsp250 + Sty250; the so-called pilot dataset), and b) a large dataset of ~12,000 samples covering almost all RS-I, RS-II, RS-III DNA samples consisting of 550 K (RS-I, II; single + duo array format) and 610 K (RS-III; quattro array format) Illumina array genotypes. In the pilot dataset also other array types have been run (including Illumina Omniexpress 2.5) allowing for comparisons and 2-step imputation strategies to create local reference data [404].

The Illumina GWAS dataset of the Rotterdam Study (with approximately 500,000 SNPs having been genotyped) also forms the basis to generate so-called “imputed” datasets derived thereof. In this process the genotypes of SNPs which have been genotyped in reference datasets (such as HapMap with ~8 million SNPs genotyped), are being estimated for all Rotterdam Study samples using the basis Illumina 500 K SNP dataset configurations in each subject. With the advent of large reference datasets becoming available based on whole genome/exome NGS, imputation activities using the Rotterdam GWAS dataset will remain an active area of development. So far, the Rotterdam Study GWAS dataset has been imputed to HapMap version 2 and 3 (with ~7.5 million resulting SNP genotypes in the Rotterdam Study dataset), and the 1000 genome dataset version 4 (with ~18 million resulting SNP genotypes in the Rotterdam Study dataset). Currently, imputations are being generated for the Genome of the Netherlands (GoNL) whole genome NGS dataset [405], and the UK10 k whole genome sequencing dataset.

The Rotterdam Study GWAS dataset is actively being used by all research lines within the Rotterdam Study as can be read under the subheadings of each research line in this review of the Rotterdam Study. In addition, it also serves as a control GWAS dataset for other research groups in- and outside The Netherlands for both SNP frequencies as well as copy number variations (CNVs), in which capacity it has been used in > 100 publications up to date. Most importantly, it has formed the start of a very successful collaboration in the CHARGE consortium (combining GWAS datasets of major epidemiological cohort studies across the world) which has >50 phenotype working groups in which almost all research lines of the Rotterdam Study are active.

### Candidate gene SNP studies

In the past, we have genotyped over 300 individual polymorphisms as part of candidate gene studies across the complete Rotterdam Study cohort using Taqman and Sequenom genotyping techniques. These mostly concern individual potentially functional SNPs per gene (e.g., ApoE), but sometimes also haplotype tagging SNPs (e.g., ESR1, ESR2, HSD11B1, fibrinogen), and also high density SNP screening (e.g., the vitamin D receptor gene). Currently, for candidate gene/SNP studies we perform look-ups in the GWAS datasets and/or perform individual SNP genotyping (e.g., SNPs not on the array, badly imputed SNPs, functional SNPs).

### RNA expression datasets

With the availability of good RNA from Rotterdam Study participants, starting with the RS-III subjects, studies have been initiated analysing the expression pattern of a single gene across samples or of the complete RNA collection in a sample (expression profiling). An expression profiling dataset has now been generated for, ±900 samples of the RS-III dataset, using the Illumina Human HT-12 v4 array containing ~48,000 probes. Subsequently, another set of ±900 samples have been subjected to RNA-sequencing (see below) so that a very rich expression dataset of in total ±1800 samples is now available. While RNA expression is known to differ between tissues, so far we only have RNA isolated from whole blood as a tissue.

### Methylation datasets

In the same samples that have RNA-expression profiles (see above) we have also generated DNA methylation profiles of ~480,000 CpG sites across the human genome using the Illumina Infinium HumanMethylation450 array. As this same set of Rotterdam Study subjects was also used for the RNA expression profiling, deep genomic studies can now take place in combination with the GWAS data and NGS data in these ~1800 subjects.

### New developments: next generation sequencing datasets

A major development in genomics studies has been the introduction in the past few years of high-throughput parallel sequencing methods (also known as next generation sequencing or NGS) which allow DNA (and RNA) sequencing at unprecedented high speed and low costs. This development has brought sequencing of whole genomes within reach of individual laboratories, rather than the large global effort that was needed to sequence the human genome at first pass. This development has led to a revival in Mendelian Genetics by solving many “cold cases” (because causal mutations could directly be found in a few samples, rather than by linkage analysis in many family samples and laborious sequencing analyses of dozens of candidate gene exons in the area). In addition, it has stimulated the cohort studies that had generated GWAS datasets in the past 5 years, to generate NGS data in part or all of their samples to find local/regional variants of interest and variants that are very rare. While whole genome sequencing (WGS) is often seen as the ultimate goal in these efforts, currently almost all labs turn to whole exome sequencing (WES) because it is much more cost-effective. In WES the parts that code for amino acids as part of the encoded proteins (i.e., the exons) are first captured from the whole genome by hybridization techniques upon which the selected parts (the exons) are then subjected to NGS. WES costs 5–10 times less euro’s than WGS (so, more samples can be done with the same amount of money) and generates interpretable findings. (note that for the vast majority of areas in the human genome we have no good idea what they signify as opposed to exons that encode functionally important parts, i.e., the parts that make up the proteins).

#### Whole exome sequencing dataset

A large grant from the NGI-sponsored Netherlands Consortium for Healthy Ageing (NCHA), allowed to generate WES NGS data for ~3000 samples by the HuGe-F facility on the Illumina HiSeq2000 sequencing machines. The samples for this experiment were selected to constitutes a random sample from the RS-I dataset. These NGS data have been generated, and variants have been called. This process is complicated and no golden standard exists, so these steps take place together with colleagues at Baylor College, the Broad Institute, and NGS facilities in Germany and Netherlands, and is likely to be repeated several times in the coming years when new software tools and data becomes available. Through a collaborative grant from the NIH Alzheimer initiative (ADSP) we will also obtain an additional ~1000 samples with WES NGS data from RS-I. Also here we collaborate with colleagues in CHARGE since NGS data have been generated in large parts of the ARIC study, the Framingham Study, and the CHS study. Within the several working groups these data are currently being analysed in relation to specific phenotypes.

From the first NGS efforts in reference datasets using WES, many thousands of novel DNA sequence variants were discovered in the exons, mostly rare to very rare and not well-covered on the existing GWAS arrays. Illumina and Affymetrix therefore decided to generate an array devoted to analyse these new exome variants called the Infinium HumanExome array. This array has been used to genotype many DNA samples in the Netherlands (sponsored by BBMRI) and across the globe and several collaborative analyses are currently using data generated with this array. In the Rotterdam Study we generated exome array genotypes of ~3000 samples of RS-I (with version 1 of this array), and this dataset is part of several collaborative efforts, including the Dutch BBMRI exome array effort (with ~35,000 collective samples), an effort in GIANT on anthropometric traits, and the CHARGE exome array effort across many different phenotype working groups (with ~60,000 collective samples).

#### RNA sequencing dataset

In a large BBMRI-sponsored collaborative effort to create a large-scale data infrastructure to work on integrative omics studies in Dutch Biobanks, the Erasmus MC HuGe-F genomics facility has generated RNA sequencing profiles of in total ±4000 individuals of six Dutch biobanks, including the Rotterdam Study. A total number of 900 RS-samples were RNA-sequenced at a depth of 30 million paired end reads. Together with colleagues at UMCG Groningen and LUMC Leiden, the dataset was QC-ed and annotated RNA-expression profiles were generated and relations between genetics, transcriptomics, and epigenetic measures are currently being analysed.

#### Microbiome 16S NGS dataset

Through applying NGS, it has recently become clear that humans carry an astonishing variety and abundance of micro-organisms (microbes, viruses, yeasts, unicellular organisms) in and on their bodies, estimated to encapsulate 100-fold more cells than a human body counts. These insights come from analysing diversity in 16S RNA sequences which are species-specific for microbes, but also from analyzing the complete microbial genomes through much deeper so-called “meta-genomics” sequencing. While microbes can be found almost everywhere in and on the body, most studies have focused on analyzing faeces/stool samples representing the microbial flora in the (last part of the) intestinal system. Sofar, this knowledge has come from very small studies with limited phenotype information outside an epidemiological setting. While the latter approach of meta-genomics is (10–20×) more expensive and generates vast amounts of data with concommittant challenges in mapping and data-analysis, most epidemiological studies focus now on establishing 16S datasets and mostly derived from stool. HuGe-F has optimized stool collection protocols to be applied in epidemiological settings (collection at home, shipment by mail), and has introduced and optimized 16S sequencing protocols (NGS of the 16S v3/v4 area). Within the Rotterdam Study RS III, we have collected ~1700 stool samples from which DNA has been isolated and which are currently subjected to 16S v3/v4 NGS analysis. For other source of microbiomes (eye, urine, mouth, skin, etc.) several pilot projects are ongoing to establish feasibility.

### New developments: integrative genomics

GWAS have identified a large number of genetic risk markers for many diseases and traits over the last decade. The Rotterdam Study has been part of many of those discoveries. However, because most variants and their proxies are non-coding, it is generally difficult to identify the causal genes. Integrative genomic studies can help to identify functional mechanisms underlying the genetic associations found. By combining genetic, transcriptomic, and epigenetic data, driving disease mechanisms can be identified. Similarly this can be done for analyzing microbiomes in this context of genetic susceptibility and genomic variables and risk factors.

Within the Rotterdam Study, novel epigenetic and transcriptomic datasets have been generated in large subsets of the study (see above) and transcriptomic data (RNAseq/array)). Part of this data is acquired within the BIOS consortium which is rainbow project funded by BBMRI-NL to create a large-scale data infrastructure and to bring together BBMRI researchers focusing on integrative omics studies in Dutch Biobanks. Using this data, we performed the largest expression quantitative trait locus (eQTL) meta-analysis so far reported in non-transformed peripheral blood samples, including replication. We identified multiple trait-associated SNPs (diabetes, cholesterol, SLE) that affect multiple *trans*-genes, and identified novel insights into these associations (PMID: 24798236). Together with collaborators from UMCG Groningen, we identified cell-specific eQTL within whole blood RNA expression data without having to sort cells [406]. By studying gene expression signatures of blood pressure, we provided new insights into molecular mechanisms underlying BP regulation [407].

As part of a second rainbowproject of BBMRI a total 2981 samples has been characterized for 232 metabolites at Brainshake’s Biomarker Analysis Platform (http://www.brainshake.fi/). The sample included all participants of RSI-4 (N = 2807) and 79 of RSII-2, 2 of RSII-3 and 87 of RS3-2.The metabolites comprise of small molecular compounds and lipoproteins subfractions.

For additional EJE references see [22, 26, 27, 149, 408–424].

## Pharmaco-epidemiologic studies

### Objective

In the Rotterdam Study, the role of drugs is studied as determinant of diseases in middle-aged and older community-dwelling individuals. This includes studying efficacy and effectiveness of drugs, as well as adverse reactions to drugs. As the drugs used in the Rotterdam Study are licensed and often on the market since several years, research focuses on determinants which modify the safety and effectiveness of widely used drugs because these often have a great impact on healthcare.

### Major findings

Below, we summarize findings over the most recent period. These findings can be distinguished into three topics of special interest, i.e. genetic and other determinants of effectiveness and adverse effects of statins; cardiac rhythm disorders and sudden cardiac death; and miscellaneous pharmaco-epidemiologic topics of interest in the Rotterdam Study.

#### Statins

The increasing incidence and prevalence of cardiovascular disease (CVD) constitutes a considerable disease burden. CVD frequently co-exists with other diseases such as type 2 diabetes mellitus (T2DM) and non-alcoholic fatty liver diseases (NAFLD), which are both strongly related to the metabolic syndrome. Statins are cholesterol-lowering drugs that are beneficial in the primary and secondary prevention of CVD. With an approximately 20–25 % reduction of the risk of major cardiovascular endpoints, these drugs have definitely entered daily clinical practice. However, some individuals may not respond adequately to statins, and non-compliance can be a problem [425]. In 2013, the American ACC/AHA guidelines on primary prevention of CVD lowered the threshold for the indication for statin treatment. According to these guidelines, we analysed in the Rotterdam Study how many people would have had an indication for statins. The ACC/AHA guideline would recommend statins for nearly all men and two-thirds of women of the Rotterdam Study, proportions exceeding those according to the ATP-III or ESC guidelines [32]. This somewhat unbelievable result shows how fast and irresistible medicalization of Western society progresses.

We investigated genetic variation that modified the efficacy and effectiveness of statins, and their risk of adverse drug reactions in clinical practice. We used the hypothesis-free genome-wide association (GWAS) approach to discover new genetic markers without a priori hypothesis of the underlying genetic variation, and the candidate gene approach to replicate genetic variation that has previously been associated with a modified statin response or occurred in a pathway that relates to statin pharmacokinetics [425]. We investigated the LDL-cholesterol lowering response to statins in a GWAS, as part of the GIST consortium including more than 40,000 statin users in both randomized controlled trials and observational studies. In this large pharmacogenetic meta-analysis, two loci at Sortilin 1 (SORT1) and SLCO1B1 were newly discovered to be associated with a stronger, and decreased LDL-cholesterol lowering response to statins, respectively. Furthermore, previously described associations with Apolipoprotein E (APOE) and Lipoprotein, Lp(a) (LPA) were confirmed, that showed a stronger and decreased LDL-cholesterol lowering response, respectively [426]. In three candidate gene studies, we investigated the influence of genetic polymorphisms on the cholesterol-lowering response to statins. First, we investigated the role of genetic variation in genes involved in cholesterol metabolism. We selected polymorphisms in these genes based on a hypothesis-free approach, and subsequently tested the most promising ones in a candidate gene analysis. We showed that two polymorphisms in two different genes, rs1532624 in the cholesteryl ester transfer protein (CETP) gene and rs533556 in the apolipoprotein A-I (APOA1) gene, were associated with a decreased cholesterol lowering response to statin therapy. The association for the CETP polymorphism was subsequently replicated in an independent population [427]. Second, we were the first study that demonstrated that two strongly linked polymorphisms in the peroxisome proliferator- activated receptor (PPARA) gene, rs4253728 and rs4823613, were associated with a stronger cholesterol lowering response to statins. Thereby, we confirmed a pharmacokinetic mechanism which was previously discovered, i.e. that these two polymorphisms were associated with significantly decreased cytochrome P450 3A4 (CYP3A4) enzyme expression and activity [428]. Third, we performed a first replication of a recent finding that the rs13064411 polymorphism was associated with increased statin-induced serum proprotein convertase subtilisin/kexin type 9 (PCSK9) concentrations, and cholesterol response to statins. PCSK9 binds to the low-density lipoprotein (LDL-) receptor, and subsequently promotes the receptor for degradation. We showed that the rs13064411 polymorphism was associated with a decreased cholesterol lowering response to statins, and this effect was stronger in women and in users of a high dose of statins [429]. The risk of MI is indirectly influenced by statins via cholesterol lowering, but is also influenced by underlying diseases, such as hypertension and T2DM. The heterogeneity in causal risk factors for a hard clinical endpoint such as myocardial infarction (MI), may affect the probability of detecting one specific gene-statin interaction and may therefore require more power than with an intermediate endpoint. The CYP3A4*22 polymorphism, previously associated with a stronger cholesterol lowering response to statins, was based on the magnitude of its effect on cholesterol, a good candidate to investigate on the outcome MI. However, we could not demonstrate significant effect modification by the CYP3A4*22 polymorphism on the effect of statins in reducing the risk of MI, neither in the independent UCP study and Rotterdam Study separately, nor in a meta-analysis of the two studies [430]. In patients with a body mass index (BMI) ≥27.5, current use of statins for more than 2 years was significantly associated with an approximately three times lower NAFLD prevalence [247].

In general, statins are safe and well-tolerated drugs, although a common ADR is myopathy, which can vary from myalgia to life-threatening rhabdomyolysis. We confirmed the previously described association between the rs4149056 c.521T>C polymorphism in the solute carrier organic anion transporting polypeptide (SLCO1B1) gene and an increased risk of developing ADRs to statins. In simvastatin users in the Rotterdam Study, we demonstrated that the rs4149056 polymorphism was associated with an increased risk of a dose decrease or switch to another cholesterol lowering drug, as indicators for ADRs. For atorvastatin users, an association was found in users with a starting dose of more than 1.00 standardized defined daily dose [431]. In another analysis in the Rotterdam Study, current use of statins was associated with lower total and (bioactive) non-sex hormone-binding globulin (SHBG)-bound testosterone levels in males. Statins decrease cholesterol production, and cholesterol is a precursor in the testosterone biosynthesis pathway. This association might be clinically relevant, given the important biological role of testosterone, the increased use of statins, and the fact that a modest average decrease in a population might hide a substantial decrease in a handful of individuals and in those with an already low testosterone level [432].

#### Drug-induced rhythm disorders and sudden cardiac death

A prolonged QT interval is an important risk factor for ventricular arrhythmias and sudden cardiac death. In an analysis in the Rotterdam Study over the 20-year period 1990–2010, a declining incidence of sudden cardiac death was observed [433]. QT prolongation can be caused by drugs, but also other factors, such as genetic variants are important for the occurrence of QT prolongation. A genetic association study of QT interval highlighted a role for calcium signalling pathways in myocardial repolarization [434]. QT prolongation is one of the most common reasons for withdrawal of drugs from the market, despite the fact that these drugs may be beneficial for certain patients and not harmful in every patient. Identifying genetic variants associated with drug-induced QT prolongation might add to precision medicine and prevent beneficial drugs from being withdrawn unnecessarily. We reassessed the association between tricyclic antidepressants (TCAs) and prolongation of the QT interval. The rationale of this study was that the heart-rate corrected QT interval (QTc) calculated with Bazett’s formula is overestimated in users of anticholinergic drugs, which also include the TCAs. In our study population, we observed that the use of TCAs was associated with a longer QTc as calculated with Bazett’s formula. However, when other correction methods were used or heart rate was included as an additional covariable in the statistical model, no association between use of TCAs and QTc was observed [435]. We also assessed the association between individual SSRIs and QTc prolongation. Of the individual SSRIs, only citalopram use was associated with a prolonged QTc in our study. Even though regulatory authorities restricted the use of citalopram to 20 mg in patients 60 years and older, participants aged 60 years and older in our study using citalopram dosage with a daily dose of more than 20 mg had a longer QTc. Although our study had a limited number of citalopram users, this study may rise questions about the safety of citalopram in high-risk patients populations (e.g., elderly) [436]. A new potentially interesting risk factor for sudden cardiac death is QT variability [437].

A second important rhythm disorder is atrial fibrillation with its high prevalence and potential cerebrovascular consequences. In the Rotterdam Study, we were able to confirm earlier work on the association between use of non-steroidal anti-inflammatory drugs (NSAIDs) and atrial fibrillation [438] and its association with hypokalemia [439]. As important potential effect modifier, we participated in a collaborative study demonstrating several new risk variants for atrial fibrillation [440].

#### Miscellaneous topics of pharmaco-epidemiologic interest

Over the last decades, antidepressant drug use increased in Western countries, including in the Netherlands. Older adults using antidepressant drugs deserve special care, as they often have a slower metabolism of drugs, impaired renal function, more co-morbidity, and more concomitantly used medications. We investigated the utilization of antidepressants in the Rotterdam Study over the period 1991–2011. The yearly prevalence of antidepressant use increased from 3.9 % in 1991 to 8.3 % of the population in 2011. The increase in SSRI use was 5.8-fold, whereas use of other antidepressants doubled and TCA use remained stable over time. The incidence of all antidepressants decreased from 23.9 to 14.2 per 1000 person-years between 1992 and 2011. The duration of a first treatment episode increased over time [441]. In a further study, we demonstrated an association between genetic variation in the ABCB1 gene and switching, discontinuation, and dosage of antidepressant therapy: results from the Rotterdam Study [442].

Several studies have been associated with haemorrhagic strokes, notably coumarins and other anticoagulants and aspirin but less is known about an association with cerebral microbleeds on MRI. In the Rotterdam Study, we investigated the association with several drugs. Use of serotonin reuptake inhibiting antidepressants was not related to presence of cerebral microbleeds [443]. Compared with never users, coumarin users had a higher prevalence of deep or infratentorial microbleeds and a higher incidence of any microbleeds, although statistical significance was not reached in the latter. A higher maximum INR was associated with deep or infratentorial microbleeds. Among coumarin users, a greater variability in INR was associated with a higher prevalence of microbleeds [444]. In stroke-free individuals, clopidogrel use was associated with a higher prevalence and higher number of microbleeds [445]. In further studies, it was demonstrated that chronic use of coumarin anticoagulants is associated with renal function decline [446]. Although thiazide diuretics are a well-known cause of hyponatremia, hypokalemia is also common [447]. Cardioselective beta-blockers were developed to prevent respiratory adverse effects, also these drugs are associated with a decreased pulmonary function [397]. In a large international consortium meta-analysis, no association could be demonstrated between pioglitazone and bladder cancer [448].

### New developments

Most new pharmaco-epidemiologic developments nowadays are associated with pharmacogenomics, proteomics and metabolomics. In an international collaborative study, we used tissue-specific quantitative interaction proteomics to map a network of five genes involved in the Mendelian disorder long QT syndrome (LQTS). We integrated the LQTS network with GWAS loci from the corresponding common complex trait, QT-interval variation, to identify candidate genes that were subsequently confirmed in Xenopus laevis oocytes and zebrafish. We used the LQTS protein network to filter weak GWAS signals by identifying single-nucleotide polymorphisms (SNPs) in proximity to genes in the network supported by strong proteomic evidence. Three SNPs passing this filter reached genome-wide significance after replication genotyping [449]. As part of methodology development, we used multiple cross-sectional assessments of depressive symptoms in a population-based study to identify potential genetic interactions with SSRIs as a model to study genetic variants associated with SSRI response. We used repeated measurement models to test multiplicative interaction between genetic variants and use of SSRIs on repeated CESD scores. Besides a genome-wide analysis, we also performed an analysis which was restricted to genes related to the serotonergic signalling pathway [450]. Furthermore, we demonstrated the potential for increased power using GEE analyses instead of cross-sectional analyses. To illustrate methods for detection of gene-drug interactions on a genome-wide scale, using repeated measures data, we conduct single-study analyses and meta-analyses across studies in three large cohort studies participating in the CHARGE consortium [451].

In the field of statistical modelling of drug exposure data, we extended our previous work on analysing drug use as a time-dependent exposure [452]. We performed a study in which the association between statins and cardiovascular endpoints was analysed with marginal structural modelling [453].

For additional EJE references see [10, 126, 128, 142, 240, 453–468].

## Imaging studies

### Objective

Biomedical imaging allows for non- or minimally-invasive assessment of structural and functional changes that may reflect specific pathology. Recent developments in image data acquisition and analysis enable to use these techniques on a large scale. The Population Imaging Unit within the Rotterdam Study aims to assess imaging biomarkers of disease in a pre-symptomatic phase at the population level. Advantages of imaging measures include that they mark early disease, can be assessed reliably and reproducibly, and are quantitative rather than qualitative which makes them more powerful than most conventional outcome measures such as clinical phenotypes.

The main imaging modalities that are currently being applied in the Population Imaging Unit are multidetector computed tomography (MDCT) and magnetic resonance imaging (MRI).

### Imaging infrastructure and storage

#### MDCT

CT imaging is performed with hospital-based 16- or 64-slice MDCT scanners (SOMATOM Sensation 16 or 64, Siemens, Forcheim, Germany), located at Erasmus MC. Scanners are operated by clinical technicians. CT images are acquired without contrast-enhancement and according to standardized protocols. Imaging data are transferred from the CT scanner to a securely backed-up research picture archiving system.

#### MRI

From August 2005 onwards, a dedicated 1.5 T MRI scanner (GE Healthcare, Milwaukee, WI, USA) is operational in the Rotterdam Study research center. This scanner is operated by trained research technicians and all imaging data are collected according to standardized imaging protocols. Changes or updates in hardware or software configuration are avoided and regular quality checks are performed to secure validity of cross-subject and cross-scan comparisons. Imaging is performed without administration of contrast agents. All imaging data are directly transferred from the scanner facility to the Erasmus MC. Data are stored on a securely backed-up research picture archiving system, using programmed scripts to check for completeness of the data received.

### Data management and processing

#### Assessment of incidental findings

All imaging data are visually evaluated within days after acquisition by trained physicians for the presence of clinically relevant incidental findings [274]. Expert radiologists are consulted for all abnormal findings and the management of clinically relevant findings is based on protocols defined by expert panels. These protocols are updated on a regular basis incorporating the current best available knowledge regarding treatment and prognosis of the various abnormalities discovered. We are furthermore actively collaborating with ethical, legal and medical stakeholders to design a framework for the management of incidental findings that can be used both nationally and internationally for population-based imaging.

#### Automated processing of MRI data

Though some measurements are still performed manually or scored visually, the majority of imaging data is now processed using semi- and fully-automated computer algorithms. The Population Imaging Unit collaborates with the Biomedical Imaging Group Rotterdam (BIGR) of Erasmus MC in the application and development of automated processing pipelines for high-throughput of large data quantities. These pipelines comprise on the one end image quality checks and procedures for non-uniformity correction, normalization and image registration and on the other end advanced algorithms to extract image features to use for analyses.

Grid architectures and networked processing pipelines are used to process the large quantities or imaging data that are acquired in the Rotterdam Study.

### Major findings

The Rotterdam Study research lines currently applying imaging within the Population Imaging Unit are those on neurological diseases and cardiovascular diseases.

#### Brain imaging (MRI)

Neurodegenerative and cerebrovascular disease are common disorders in the elderly that exert a large influence on brain functioning. Identifying underlying pathology in a preclinical state may help to recognize persons at risk, assess determinants of disease and develop preventive measures. Main objective for the Population Imaging Unit with respect to brain imaging is therefore to identify and quantify brain imaging biomarkers that mark the development of neurodegenerative and cerebrovascular disease.

From August 2005 onwards (RS-II-2 and onwards), brain imaging in the Population Imaging Unit is performed in all study participants without contra-indications in the context of the Rotterdam Scan Study. The current structural scanning protocol includes 4 high-resolution axial sequences (3D T1-weighted; 2D PD-weighted; 2D FLAIR; and 3D T2* GRE), 2D phase-contrast imaging, and diffusion tensor imaging (DTI). Total scanning time for these sequences amounts to approximately 30 min. Currently, over 5,800 unique brain MRI scans and over 6,300 follow up scans have been acquired with this protocol. Starting 2012, functional imaging has been incorporated in the MRI protocol in the form of a resting-state fMRI sequence (8 min), which was added to the existing structural scans. To date, rs-fMRI scans have been acquired in over 2,000 individuals and this is ongoing.

Fully automated methods are applied to quantify atrophy of brain tissues and structures and the severity of white matter lesions [294, 469, 470]. Automated hippocampal segmentation has been successfully applied on multi-scanner acquired MR images (on scans acquired in the Rotterdam Scan Study in 1995 [288] and follow up examinations in 2006), showing that a decline in hippocampal volume over a 10-year follow up period predicted onset of clinical dementia [471]. Apart from focusing only on supratentorial brain structures, we have also incorporated segmentation of the cerebellar volume into our automated processing pipelines, enabling us to study the cerebellum in aging.

Phase-contrast imaging allows for assessment of blood flow in the carotids and basilar artery. This yields measures of total brain perfusion [472], which when lower was found to be relate to worse cognition, an association that is mediated by brain atrophy [473]. More recently, we used our longitudinal imaging data to address the ongoing debate whether reduced perfusion causes brain atrophy or vice versa, and found that brain volume reduction relates to decrease in perfusion over time, presumably through lower metabolic demand [474].

The 3D T2* GRE sequence that we use was specifically developed to increase the conspicuity of cerebral microbleeds [475]. With this optimized sequence, we found that microbleed prevalence gradually increases with age, from 6.5 % in persons aged 45 to 50 years to 35.7 % in participants of 80 years and older; and that overall, 15.3 % of all subjects over the age of 45 years has at least 1 microbleed; a much higher prevalence than was reported before [476, 477]. We found supportive evidence that deep or infratentorial microbleeds reflect arteriolosclerotic angiopathy, whereas strictly lobar microbleeds are caused by cerebral amyloid angiopathy [476, 477]. We furthermore demonstrated that incidence of microbleeds over a 3-year time interval is high and that risk factors for new microbleeds again differ according to microbleed location, in line with our findings regarding prevalent microbleeds [478]. These findings impact research into the causes of cerebral amyloid angiopathy, as well as fuel the ongoing discussion about the safety of antithrombotic therapy in persons with microbleeds [444, 479, 480]. Our most recent studies focus on the interplay between haemorrhagic and ischemic imaging markers [481], pointing towards a shared etiologic pathway. This is further supported by our finding that microbleeds relate to an increased risk of stroke, both ischemic and haemorrhagic, over a mean follow up of five years [482]. Diffusion tensor imaging (DTI) allows the assessment of the microstructural integrity of white matter. White matter microstructure loses its integrity with increasing age, but this can largely be explained by presence of white matter atrophy and white matter lesions [483]. Nevertheless, the microstructural integrity in the normal appearing white matter and in white matter lesions relates to cognitive function regardless of concurrent macrostructural changes, emphasizing the importance of the microstructural integrity of white matter [484]. Also, we found that white matter changes in normal appearing white matter are present and can be quantified on diffusion tensor imaging before white matter lesions actually develop. This suggests that white matter lesions develop gradually, and that visually appreciable lesions are only the tip of the iceberg of white matter pathology. We developed within the Rotterdam Study a method for fully automated tractography of over 20 major white matter tracts, yielding tract-specific measures of white matter integrity, enabling a more in-depth exploration of structural integrity in relation to functional processes in aging [485]. Using these tract-specific measures, we could demonstrate widespread deterioration of white matter integrity with age, which nevertheless showed variations across tract groups, with the motor system being relatively spared [486]. We are currently extending this work to assess longitudinal changes in white matter integrity and tract-specific influences on cognitive function.

#### Imaging of atherosclerotic calcifications (MDCT)

Main objectives with respect to imaging of vascular calcifications are to investigate distribution of and risk factors for atherosclerotic calcifications in the general elderly population and to study prognosis associated with calcifications in different vessel beds.

From September 2003 until February 2006, all participants from RS-I-4 and RS-II-2 who completed a center visit were invited to a MDCT scan of the coronary arteries and a second scan of the aortic arch and carotid arteries. A total of 2521 participants (response rate 79 %) were scanned. The cardiac scan reached from the apex of the heart to the tracheal bifurcation. The second scan reached from the aortic arch to the intracranial circulation. Images were analysed by trained reviewers and calcification in the different vessel beds (coronaries, aortic arch, extracranial and intracranial carotids) were quantified using the Agatston score [487].

As expected, we found that calcification load was higher overall in men compared to women, though aortic arch calcification was more prevalent among women [488]. Age and current smoking were found to be the strongest independent risk factors for arterial calcification [489]. Furthermore, strong and graded associations of prevalent stroke were found with carotid artery, aortic arch and coronary artery calcification, independent of cardiovascular risk factors [490]. When vessel calcification was studied in relation to vascular brain disease (non-invasively imaged with MRI), we found that larger intracranial carotid calcification load related to larger white matter lesion load, and that larger extracranial carotid calcification load related to the presence of cerebral infarcts, independently of ultrasound carotid plaque score. This suggests that calcification of atherosclerotic plaque yields other information in addition to merely the presence of plaques. This importance of vessel calcification is further corroborated by our recent finding that larger calcification volumes are associated with worse cognitive performance in the general population [276].

Despite extensive research on the identification of lifestyle- and environmental cardiovascular risk factors, a large part of the variability in the total burden of atherosclerosis remains yet unexplained. This inevitably indicates that other, unknown factors also considerably contribute to the development of atherosclerosis. During the last years, it has become clear that genetic factors may play an important role in the development of atherosclerosis. We determined whether previously identified genetic loci for coronary calcification were also associated with calcification in other locations than the coronary arteries Indeed, we found that the genetic basis for aortic arch and carotid artery calcification largely overlaps with that of coronary artery calcification. However, we found that the genetic variants contributed differentially to the amount of atherosclerotic calcification in these vessel beds. This suggests that also on the genetic level, differences in the etiology of atherosclerosis across vessel beds exist. Additionally, we also investigated the genetic basis of atherosclerosis using the genetics of strong risk factors of atherosclerosis, i.e. serum cholesterol levels and blood pressure and demonstrated that both serum lipids and blood pressure share a genetic predisposition with the formation of atherosclerosis in multiple vessel beds.

Worldwide, intracranial atherosclerosis is one of the leading causes of stroke, yet it is understudied in population of white descent. We therefore focused specifically on the prevalence and risk factors of intracranial internal carotid artery calcification in our population, and found that the overall prevalence of intracranial atherosclerosis was as high as 82.2 %. Conventional cardiovascular risk factors are associated with intracranial atherosclerosis, but risk factor profiles differed between men and women, with excessive alcohol intake and smoking were strong risk factors in men, whereas diabetes and hypertension were in women. We were the first to demonstrate in this general population-setting, that intracranial atherosclerosis is also a major cause of stroke in whites [491]. Even after adjustments for large-artery atherosclerosis in the aortic arch and the carotid artery bifurcation, this relationship was firmly present. Moreover, we found that intracranial atherosclerosis contributed to 75 % of all strokes, whilst for aortic arch atherosclerosis or atherosclerosis in the carotid artery bifurcation this was 45 and 25 %, respectively. Altogether, these findings emphasize that existing location-specific differences in atherosclerotic burden indeed translate into differences in risk of subsequent cerebrovascular disease.

With our study on atherosclerosis and the risk of dementia, we further established the role of atherosclerosis in the etiology of dementia [276]. Most importantly, we found that systemic atherosclerosis was associated with a higher risk of dementia and cognitive decline. Actually, this suggests that generalized atherosclerosis, which probably is a better reflection of one’s vascular status rather than localized atherosclerosis in a single vessel bed, associates with dementia.

#### Carotid plaque imaging (MRI)

Carotid wall thickening and atherosclerosis are highly prevalent at older age and are considered a major cause of cerebrovascular events [268]. Carotid atherosclerotic plaque constituents such as lipid core and haemorrhage, so-called “vulnerable” components, are considered important factors in development of clinical neurological events [492]. With MRI, it is possible to separately identify these plaque components [493]. Main objectives with respect to carotid imaging in the Rotterdam Study are to investigate distribution of and risk factors for carotid plaque components in the general elderly population and to study prognosis associated with specific carotid plaque composition.

From October 2007 onwards, all participants with carotid wall thickening of 2.5 mm or larger on ultrasound (approximately 25 % of the Rotterdam Study population) are invited for carotid MRI. Imaging is performed with a bilateral phased-array surface coil (Machnet, Eelde, the Netherlands), stabilizing subjects in a custom-designed head holder to reduce motion. The imaging protocol consists of a series of high-resolution MRI sequences to image the carotid bifurcations on both sides: a PDw Fast Spin Echo (FSE) Black-blood (BB) sequence; a PDw-FSE-BB with an increased in-plane resolution; a PDw- Echo Planar Imaging (EPI) sequence and a T2w-EPI sequence; and 2 three-dimensional (3D) sequences: a 3D-T1w-Gradient Echo (GRE) sequence; and a 3D phased-contrast MR-Angiography. Total scanning time amounts to approximately 30 min.

Plaques are reviewed by trained raters for the presence of three different plaque components (calcification, haemorrhage and lipid core). Furthermore, carotid plaque size is quantified by obtaining maximum carotid wall thickness and degree of luminal stenosis using the NASCET criteria [494] on the PDw-FSE images. Postprocessing techniques aimed at automated quantification of plaque volume and identification of different plaque components are currently being developed.

So far, close to 2000 participants underwent a complete carotid MRI scan and data are currently being analysed. There is a complete overlap between carotid and brain MRI participants, allowing for the investigation of carotid plaque constituents in relation to brain imaging markers. In the first 1,006 scans, we found that intraplaque haemorrhage and lipid core were present in almost 25 % of plaques, respectively, and occurred simultaneously in 9 % of plaques. Different risk factors are associated with these plaque components: hypertension (and in particular high pulse pressure) and current smoking were risk factors for the presence of intraplaque haemorrhage, and hypercholesterolaemia was the only risk factor for lipid core presence.

Ischemic strokes are more often diagnosed in the left hemisphere than in the right, therefore we hypothesized that asymmetry in carotid atherosclerosis may play a role in this observation.

We indeed demonstrated that plaque prevalence, severity and composition are not equally distributed among the left and right carotid arteries [495]. Left-sided plaques were slightly thicker than the contralateral side and were predominantly composed of IPH and fibrous tissue. These findings suggest that atherosclerotic plaques on the left are more vulnerable than on the right.

Using the strength of both CT and MRI data in a subsample of our population, we were able to assess the relationship between calcification, intraplaque haemorrhage and lipid core within the carotid atherosclerotic plaque [496]. Plaques with a higher load of calcification contain more often haemorrhagic components, but less often lipid core. These findings suggest that a higher load of calcification does not imply that a plaque is more stable, and urge for prospective studies investigating the interrelation of these different plaque components with regard to future cerebrovascular events.

### New developments

As also mentioned above, focus has shifted from purely structural imaging to also including functional imaging data, by incorporating resting-state functional MRI into the brain imaging protocol. Changes in the intrinsic activity of resting-state networks are presumed to represent alterations in functional brain connectivity and may mark neurodegeneration in an early, presymptomatic stage. Our initial studies will focus on the relation between functional brain connectivity and established structural markers in age-related brain diseases such as hippocampal volume, white matter lesions and microbleeds. In a later stage, we will investigate whether functional connectivity can be used an early imaging marker for dementia, by itself or in combination with other imaging markers and risk factors.

Regarding structural imaging markers, an emerging potential marker is Virchow–Robin (VR) spaces, spaces filled with interstitial fluid that surround the blood vessels in the brain and which can be dilated. Despite increasing literature on these dilated VR spaces, a major limitation of current research is the lack of a robust and generalizable rating method on MRI. Within the Rotterdam Study, we developed a novel rating method for VR spaces, which we successfully applied in 2 population-based studies, encompassing 3 different scanning protocols. We are now using this rating method to explore determinants and consequences of dilated VR spaces in our general aging population, as well as in a larger consortium of other clinical and population studies (www.uconsortium.org).

Besides ever-increasing advances in imaging hardware, software and sequence design, major advances in the short and long run are to be expected from (fully) automated image analysis. Computer processing of images will enable to make fully use of all information contained within the image, introducing new imaging biomarkers. Besides, the vast amount of imaging data that are acquired in population-based studies like the Rotterdam Study renders visual assessment or manual measurements virtually impossible, strengthening the need for (fully) automated methods of data extraction and analysis.

For additional EJE references see [13, 400, 497–500].

## Otolaryngological diseases

### Objectives

Otolaryngological research in the Rotterdam Study focuses on the frequency, etiology and consequences of auditory and vestibular disorders. We are mainly interested in dysfunctions located in the labyrinth of the inner ear, expressed by cochlear hearing loss and a deviant vestibulo-ocular reflex (VOR) for fast head movements. Etiology of both peripheral disorders will be studied, possibly revealing one or more common risk factors as both systems are connected and have similar sensory mechanisms. Additionally, we will investigate the consequences of reduced sensory input on brain development and function.

### Methods

Data on both hearing and vestibular function have been collected in the cohorts RS-II-3and RS-III-2. Hearing data has also been collected in RS-I-5. Hearing loss is assessed at both ears by performing pure-tone audiometry in a sound proof room. Hearing thresholds are determined with headphones at frequencies 0.25, 0.5, 1, 2, 4 and 8 kHz. To distinguish between cochlear and middle-ear pathology, also bone-conduction thresholds are measured at frequencies 0,5 and 4 kHz. Additionally, speech perception in noise is tested at the better ear, using a validated triplet digit test [501] with speech-shaped noise at a fixed presentation level. The ability to understand speech in noise is a functional measure that includes both sensory and central aspects of the auditory system.

Peripheral vestibular function is assessed by The Head Impulse Test (HIT), which measures the vestibule-ocular reflex (VOR) for a number of sudden head movements initiated by the tester [502]. Gain and delay are the main parameters that will be used to quantify vestibular function. An advanced infrared video-oculography system is used to track the eye movements simultaneously with the position of the head, enabling VOR recordings, even for fast head movements. Additionally, central vestibular processing is assessed by measuring the VOR during repetitive slow head movements at frequencies 0.5, 1 and 2 Hz while fixating the eyes at a projected visual target. The oculomotor function is tested by eye tracking of a moving target at frequencies 0.5, 1 and 2 Hz, while the head is fixated (the smooth pursuit).

The general interview contains ten questions related to hearing and balance problems. In case of hearing-aid use, the participant has to answer five additional questions of the International Outcome Inventory of Hearing Aids (IOI-HA) [503]. In case of frequent tinnitus, ten additional questions of the Short Tinnitus Handicap Inventory (THI-S) are added [504].

### Determinants of interest

One of our main goals is to find medical and genetic factors that are associated to age-related hearing loss. Age-related hearing loss is a common disorder that deprives older people of key sensory input. It leads to social withdrawal [505] and is even been found to be independently associated with poorer cognitive functioning and incident dementia [506, 507]. Still, little is known about the mechanisms that are responsible for developing hearing loss and the way it affects general cognitive functions within the elderly population. Determinants of interest are genetic factors, cardiovascular disease, use of medication, endocrine diseases and neuroepidemiological factors. Analyses of genetic risk factors will be performed in close collaboration with other groups within the CHARGE consortium.

Even less is known about age-related vestibular dysfunction. Problems related to balance and dizziness are frequently reported in elderly people, but there is still uncertainty about the prevalence and type of vestibular dysfunction that may cause these problems [508]. This is one of the first large cohort studies in which vestibular function will be measured. The aforementioned determinants for hearing will also be studied for the vestibular system. Additionally, the relation between hearing and peripheral vestibular function will be extensively investigated.

### Major findings

In order to have sufficient statistical power and age coverage we combined the data collection of RS-II-3 and RS-III-2. First data analyses on hearing have been completed and main outcomes will be published in near future. In general, hearing loss is highly prevalent within the tested group. We found a hearing loss of 20 dB HL or higher in more than 50 % of the people. The amount of hearing loss is higher in men than in women, although the difference is smaller than previously reported and presented by the current clinical standards. As expected, hearing loss is strongly associated with age and leads to a poorer understanding of speech in background noise.

## Management

The Rotterdam Study is directed by a Management Team comprising the scientific principal investigators Sarwa Darwish Murad (PI Hepatic diseases), Cornelia van Duijn (PI Genetic epidemiologic studies), Oscar Franco (PI Cardiovascular diseases), André Goedegebure (Otolaryngological diseases), Albert Hofman (chairman, PI Rotterdam Study), Arfan Ikram (PI Neurological diseases), Caroline Klaver (PI Ophthalmic diseases), Tamar Nijsten (PI Dermatological diseases), Robin Peeters (PI Internal Medicine), Bruno Stricker (PI Pharmaco-epidemiology), Henning Tiemeier (PI Psychiatric diseases), André Uitterlinden (PI Genomic studies), and Meike Vernooij (PI Population Imaging); and Jan Heeringa, MD, PhD, study coordinator, Eric Neeleman, head IT, and Frank van Rooij, MSc, head data-management.

## Emeritus principal investigators

The following persons are Principal Investigator Emeritus of the Rotterdam Study:

Frank van den Ouweland (PI Internal Medicine 1990–1992), Diederick Grobbee (PI Cardiovascular diseases 1990–1996), Albert Hofman (PI Neurological diseases 1990–1996), Paulus de Jong (PI Ophthalmic diseases 1990–2005), Huibert Pols (PI Internal Medicine 1993–2006), Monique Breteler (PI Neurological diseases 1997–2010), Gabriel Krestin (PI Population Imaging 1998–2010), Johannes Vingerling (PI Ophthalmic diseases 2005–2010), Jacqueline Witteman (PI Cardiovascular diseases 1997–2011), Ernst Kuipers (PI Internal Medicine 2007–2013), Harry Janssen (PI Hepatic diseases 2007–2013).
